# An intelligent algorithm for energy efficiency optimization in software-defined wireless sensor networks for 5G communications

**DOI:** 10.1371/journal.pone.0301078

**Published:** 2024-06-20

**Authors:** Kemal Gökhan Nalbant, Suliman A. Alsuhibany, Asma Hassan Alshehri, Maha Hatira, Bong Jun Choi

**Affiliations:** 1 Faculty of Engineering Architecture, Department of Software Engineering, Istanbul Beykent University, Sariyer, Istanbul, Turkey; 2 Department of Computer Science, College of Computer, Qassim University, Buraydah, Saudi Arabia; 3 Department of Computer Sciences, College of Computer Engineering and Sciences, Prince Sattam bin Abdulaziz University, Al Kharj, Saudi Arabia; 4 Faculty of Engineering and Information Technology, Taiz University, Taiz, Yemen; 5 Islamic University Centre for Scientific Research, The Islamic University, Najaf, Iraq; 6 School of Computer Science and Engineering, Soongsil University, Seoul, South Korea; TU Wien: Technische Universitat Wien, AUSTRIA

## Abstract

Wireless communications have lately experienced substantial exploitation because they provide a lot of flexibility for data delivery. It provides connection and mobility by using air as a medium. Wireless sensor networks (WSN) are now the most popular wireless technologies. They need a communication infrastructure that is both energy and computationally efficient, which is made feasible by developing the best communication protocol algorithms. The internet of things (IoT) paradigm is anticipated to be heavily reliant on a networking architecture that is currently in development and dubbed software-defined WSN. Energy-efficient routing design is a key objective for WSNs. Cluster routing is one of the most commonly used routing techniques for extending network life. This research proposes a novel approach for increasing the energy effectiveness and longevity of software-defined WSNs. The major goal is to reduce the energy consumption of the cluster routing protocol using the firefly algorithm and high-efficiency entropy. According to the findings of the simulation, the suggested method outperforms existing algorithms in terms of system performance under various operating conditions. The number of alive nodes determined by the proposed algorithm is about 42.06% higher than Distributed Energy-Efficient Clustering with firefly algorithm (DEEC-FA) and 13.95% higher than Improved Firefly Clustering IFCEER and 12.05% higher than another referenced algorithm.

## 1 Introduction

Fifth Generation (5G) technology represents a significant advancement over all prior mobile generation networks and is a cornerstone of the digital transformation. Three new services, including Extreme Mobile Broadband (eMBB), are available to end users using 5G. In addition to many other features, it provides increased bandwidth, ultraHD streaming movies, virtual reality and augmented reality (AR/VR) media, high-speed internet access, and minimal latency. Massive machine type communication, or eMTC, offers broadband and long-range machine-type communication at a very low cost and with minimal power usage. For Internet of Things applications, eMTC offers mobile carriers a high data rate service, low battery consumption, and wider coverage with fewer complicated devices. With its exceptionally fast speed, high throughput, low latency, improved reliability and scalability, and energy-efficient mobile communication technology, 5G offers a limitless internet connection at your leisure, anytime, anywhere [1,2].

Software-defined network (SDN) is a strategy to enable programmatically efficient network design and to facilitate network administration, hence improving network performance and monitoring [3]. It opens up a world of new creative applications, from traffic engineering to data centre virtualization, fine-grained access control, and more, by separating the data and control planes [4]. It is a strong option for 5G networks as well because it has a demonstrated benefit in several commercial networks [5,6].

A wireless sensor network (WSN) typically uses a base station and multiple sensor nodes. Due to its low deployment cost and great benefits, WSN has gained rapid development and wide popularity [1–3]. However, WSN suffers from limited network resources, inflexible network management, and partial efficiency of routing algorithms [4–6]. To solve the above problems, SDN based Wireless Sensor Networks (SDWSN) [7–10] apply software-defined technology to WSNs by separating data and control planes. The SDWSN improves network flexibility through centralized management and programmability, supports high-speed heterogeneous connectivity, flexible and efficient detection, dynamic and reliable routing, and improves WSN utilization [11–13].

Network energy efficiency is still a major obstacle to WSN deployment in SDN [14–17]. Since the original routing method is no longer fully suitable for WSNs, it is important to develop a routing strategy that considers the characteristics of SDWSNs [18–20]. Various routing algorithms such as Low Energy Adaptive Clustering Hierarchy (LEACH) [21], Distributed Energy-Efficient Clustering (DEEC) for heterogeneous WSNs [22], Stable Election Protocol (SEP) [23], and other clustering routing algorithms are deployed in SDWSN. The SDWSN exhibits strong scalability and robustness because its cluster routing algorithm divides sensor nodes into multiple clusters. Each cluster has a cluster head that collects information from its members and sends it to the base station. LEACH randomly selects cluster heads to balance network load. However, polling the cluster heads frequently consumes power [24]. When choosing cluster heads, DEEC considers the remaining energy of the nodes, prolonging the network lifecycle and increasing the data transmission delay.

The clustering step of the clustering routing method, an approach to solving difficult optimization problems by modeling the behavior of groups, has recently been improved by applying some clever optimization algorithms. The Artificial Bee Colony Algorithm (ABC) [25], the Firefly Algorithm (FA) [26], and the Genetic Algorithm (GA) [24] are examples of intelligent optimization algorithms. The GA local search performance is poor despite its fast convergence time. The FA has fewer parameters than GA. Global search and convergence speed are better for the ABC method. Methods that are easy to integrate with clustered routing algorithms include the FA and ABC algorithms. Intelligent optimization techniques suffer from poor solution accuracy, convergence to locally optimal solutions, and limited search space.

The main contributions of this paper are as follows.

1. Establish the network model and energy consumption model of SDWSN.
2. A hybrid optimization algorithm (optimized algorithm based on the firefly algorithm, gravity search algorithm, and biogeography optimization, FGB) was designed. To improve the capability of a worldwide search for the best solution, a location update approach based on population diversity is first presented and is based on FA. Then, based on the hybrid gravitational search algorithm (GSA) and biogeographic-based optimization (BBO), the ability of local search to find the optimal solution is optimized. The algorithm improves the performance of optimization procedures that find approximate global solutions.
3. A distributed high-efficiency entropy energy-saving clustering routing algorithm for SDWSN is designed (DHEEC). This algorithm considers energy entropy when selecting cluster heads, which is more suitable for energy updates in SDWSN. At the same time, FGB is used for cluster head selection to speed up the rotation of cluster heads.
4. Through experimental simulation and analysis, compare FGB with other algorithms to verify its performance on standard functions. Comparing DHEEC with other algorithms, it prolongs the network lifetime and improves energy utilization.

The remaining of this this paper is organized as follows. In Section 2, the literature review is discussed in detail. In Section 3, the proposed system model is described and the analytical formulations are discussed. In Section 4, the hybrid optimization algorithms are explained. In Section 5, the high entropy energy saving clustering routing algorithm is described. In Section 6, the simulation experimentations and comparative performance evaluation is discussed. Finally, Section 7 concludes the paper.

## 2 Related work

This section mainly focuses on two aspects of the network clustering routing algorithm and the intelligent optimization algorithm.

A three-stage heterogeneous network model for WSN is proposed in [27]. The model uses a threshold function and weighted election probabilities to select cluster heads and capture network heterogeneity. Based on the evolutionary approach for LEACH protocol optimization (LEACH-O) [28], both WSN lifetime and energy efficiency are improved. The Enhanced Adaptive Distributed Energy Saving Cluster (EADEEC) algorithm [29] based on DEEC improves cluster stability, node survival time, and data transmission. The authors in [30] uses particle swarm optimization (PSO) to estimate the relative positions of cluster heads and base stations, developed mobile paths for base stations based on the ABC traversal path approach, and reduced sensor node consumption power.

The clustering phase of the clustering routing method uses a population optimization algorithm. In [31], an energy-efficient heterogeneous WSN routing technique based on Improved Firefly Clustering (IFCEER) was developed. However, it has poor solution accuracy and slow convergence are also problems. The WSN routing method based on FA for optimizing fuzzy C-means (FFACM) is a good reference to prevent the algorithm from tuning to local optima [32]. This is due to incorrect initial placement of the clustering centers. The authors in [33] considered the Bayesian PSO algorithm that is based on the Bayesian principle probability strength function for parameter tuning. However, especially complex multi-minimal problems face local optima. The Hybrid Firefly and Particle Swarm Optimization Algorithm (HFPSO) [34] checks the current global best fit value and decides to start the search process. In [35], a hybrid firefly algorithm with particle swarm optimization (HFAPSO) is proposed. This algorithm uses the HFPSO for optimal cluster head selection on LEACH-C and improves the FA’s global search ability. Reference [36] applied FA to a mixed-state logic model with an energy parameter to detect distributed denial-of-service (DDoS) attacks. Reference [37] used FA and BBO for feature selection in software product lines. In [38], the Gray-Wolf optimization (GWO) algorithm was combined with PSO to significantly reduce the computational time required to implement the algorithm.

The LEACH-G protocol was proposed in [39], where the calculation of the ideal cluster head size is based on area and number of nodes. The enhanced LEACH protocol should theoretically outperform the original LEACH in terms of network robustness and power consumption. The R-LEACH is a clustering strategy designed based on the remaining energy of nodes and the maximum number of cluster heads [40]. The probability threshold is redefined by the ideal number of cluster heads to improve the network speed and reduce the average power consumption. To achieve network load balancing, reference [41] provides a power-aware routing algorithm that creates timeouts according to the remaining power of a node and controls how often a node is elected as cluster leader. Each node determines its own attribute value based on the distance from the base station and the amount of energy left over, and based on this, it competes with neighboring nodes to obtain the best cluster head selection. Reference [42] suggested a distributed energy-saving clustering routing method. In [43], the authors found that if each cluster region remains fixed after establishment, the cluster head election is performed in each cluster region based on the residual energy factor, and if the cluster reaches the head energy, proposed an improved low-energy fixed clustering algorithm. If the threshold is below, a node from the cluster her members is randomly selected for the next voting round. This algorithm is efficient and prolongs network life. Reference [44] proposes a modified DEEC (MDEEC) algorithm that extends the network lifecycle but increases the amount of data received by the base stations. Additionally, a low-power communication mechanism was established within the cluster to allow nodes with residual power above a threshold to compete as cluster head candidates. Reference [45] proposed an improved protocol called LEACH-LOMUC, which selects candidate cluster heads based on remaining energy, size of cluster heads and distance between clusters, cluster head and base station transmission line, which effectively saving network energy. This protocol overcomes the shortcomings of the LEACH algorithm, which does not take into account the residual energy, the distribution of the cluster head, and the direct communication between the cluster head and the base station. Reference [46] proposed a distributed clustering routing protocol based on dynamic partition load balancing to solve the “hot zone” problem caused by uneven load on the nodes. Distributed clustering routing protocol determines different cluster radius based on the distance between the cluster head and base station, thus prolonging the life of the network. Reference [47] suggested that a highly efficient relay and relay node selection scheme designed for farmland environments to solve the problem of high power consumption caused by direct communication between the cluster head node is located far away from the base station and the receiver node. The node’s residual energy, the density of the network architecture, and the distance are carefully considered when choosing a cluster leader. The forwarding methods in different communication models are described one by one, starting with the distance between the candidate forward node and the sink node during the relay node selection process. The energy efficiency of the network is increased when energy balance is achieved. By setting the fit function to remaining energy and the distance from the base station, references [48,49] improved the weights of the first three wolves in the original GWO based on price suitable treatment. They also designed an improved algorithm based on the Physical Interest-Based Advanced GWO (FIGWO), which allows for optimal election of cluster leaders. Reference [50] has improved the particle swarm optimization algorithm based on adaptive learning factors, determined the adaptive function based on the node energy factor and the position equilibrium factor, and proposed two transmission methods. The throughput (single-hop and multi-hop) is optimized according to the distance between the cluster head and base station, while balancing the energy consumption of the nodes. Reference [51] proposed a heterogeneous clustering technique based on ant colony optimization to solve the problem of heterogeneity. This algorithm used the concept of pheromone update primacy to optimize the path quality and significantly improve the network quality. The optimal cluster head is selected by evaluating the relationship between the random number and the attribute and the weight parameters are optimized based on the GA and the simulated incubation algorithm. Reference [52] proposed a low-power routing protocol, where the node attribute is defined around the distance between the node and the base station and the remaining power factor. Reference [53] proposed a hybrid heterogeneous clustering routing protocol based on fuzzy logic theory and ant colony optimization. Based on the ant colony optimization method, the heterogeneous cluster regions are created, allowing efficient transmission of monitoring data from the cluster head to the base station, thereby effectively reducing the power consumption of the network. For the purpose of balancing the energy consumption of the network, showing the relationship between the cluster head ratio and the network size, and designing the suitable function for the particles of the swarm optimization algorithm on the energy remaining and node density, the improved filtering based on the PSO algorithm is proposed in [54], which greatly prolongs the life of the network. Using a GWO and a unique fit function, reference [55] proposed a new clustering protocol to predict the actual power consumption of multiple clustering schemes, giving allows reuse of a particular clustering method. A two-way communication network is established between the peripheral cluster leader and the receiving node to prevent premature death of nodes far from the base station. This network effectively balances the power consumption between the cluster head and the relay node, while reducing the overall network cost.

Reference [56] proposed a centralized clustering technique (LEACH-C), which would improve the existing LEACH protocol. The base station selects the best cluster head in the cluster head election step using a simulated annealing process. To solve the problem of random selection of cluster leaders using the LEACH protocol, the LEACH-C takes into account the node’s position and remaining energy when selecting the cluster leader. However, due to one-hop communication between the cluster heads and base stations, the energy consumption of cluster heads far from the base station will increase, eventually leading to their premature collapse and network lifetime. Reference [57] proposed a centralized cluster routing protocol in which the base station groups the nodes for each round of cluster leader election using averaging technique and the GWO selects the best cluster leader for each cluster. By improving the cluster structure, it effectively increases the lifetime of the network. However, because this protocol does not take into account the routing between clusters, the cluster head located far from the base station will die prematurely due to high power consumption, reducing the lifetime of the network. References [58] proposed a centralized clustering routing system based on K-Means. The base stations use K-Means clustering to create the network clusters during network startup. The nodes disperse the cluster heads and remaining energy in each cluster. The K-Means clustering technique is sensitive to the starting point and simple to converge to the local optimal point, which has an impact on the clustering effect. However, it prolongs the life of the network to some extent. Furthermore, the protocol does not take routing between clusters into account, and the single-hop technique increases the energy consumption of the cluster head when it is far from the base station. This places constraints on the system. The ABC-SD protocol, which uses the artificial swarm method to solve the clustering and routing problems, has been presented in [59]. By taking into account the remaining energy of the nodes and neighboring data, this protocol creates an efficient matching function that is used to evaluate the results of the artificial swarm method. The clustering problem can be solved depending on the quality of the solution. The protocol builds routing between clusters based on the loss function after considering the energy efficiency and the number of hops in the routing hop between the clusters. The ABC-SD effectively extends the network lifetime and increases the network throughput by implementing centralized clustering and distributed route mix generation techniques. However, the load balancing between cluster heads, which affects network performance, is not taken into account by this protocol. A technical improvement based on the clustering routing protocol of the PSO algorithm has been proposed in [60]. A new matching function is designed by considering the redundant energy and the geographical location of the node in the grouping step to select the group leader who is near the base station and has a high level of excess energy. This fitness function is then used to evaluate the quality of the solution generated by the swarm algorithm. Each cluster head is paired with a relay node in the routing step between the clusters to balance the network energy consumption. The network lifetime is effectively extended by this protocol. However, there is no guarantee that the selected forward and cluster leaders are the current optimal option due to some features of the swarm optimization process itself. Reference [61] suggested a software-defined network routing method based on fuzzy logic. It was used as the basis for implementing the Fuzzy Topology Discovery Protocol (FTDP). It prolongs the life of the network and reduces the packet loss rate. To increase the lifetime of the network and reduce the latency, reference [62] developed an intelligent routing method based on the ABC algorithm that maps potential routing links as food sources and takes into account energy and delay variables. The Fuzzy Ant Colony Optimization Routing (FACOR) was developed by [63] by incorporating the ant colony algorithm into the routing protocol, evaluating the link viability by using fuzzy calculations based on energy, distance and connectivity, then choose the optimal link. A metaheuristic search routing protocol based on GA and mutation operator is presented in [64]. To develop effective routing and load balancing strategies, reference [65] introduced the load balancing and routing strategies using the Glowworm swarm optimization approach (LBR-GSO). Using the properties of a dynamic network, reference [66] suggested an improved ant colony algorithm (IACO), which can be used to generate sensor and rule node propagation functions. The network is clustered heterogeneously in [67], and the smaller the distance from the sink node, the smaller the cluster size. This reduces communication power loss in the cluster and provides the cluster main node with more power to transfer data between clusters, balancing the network power consumption and successfully avoiding the main problem. Reference [68] proposed a WSN hierarchical routing technique based on fuzzy cluster C-means and swarm intelligence (FCM-SI). The optimal cluster leader is selected using an artificial swarm algorithm, and then establishes a multi-hop path to the base station using the ant colony method. Although the power consumption and load balancing of the network are taken into account, the effect of the random starting cluster center of the fuzzy C-means algorithm is not taken into account. This can easily lead to locally optimal clustering, which will affect the entire network. An energy-aware multi-hop routing (EAMR) method is proposed in [69]. During the setup phase, a permanent cluster is created by selecting the initial cluster leader, selecting the first relay nodes, and determining the cluster membership of the remaining sensor nodes. The data is passed through the relay node during this phase, thus balancing the load on the cluster. However, the power consumption of the fixed burst far away from the base station is significantly higher than that of the fixed burst near the base station as the amount of data transmission increases. The clusters cannot evenly distribute network load. Reference [70] proposed effective and reliable routing algorithm based on dempster-shafer proof theory (DS-EERA) in which the attribute index is established as proof, using weighted method and the number of entropy to determine the weight of the index. Then, apply the merge rule of the theoretical DS proving to the underlying probability distribution function of each merged index value and the next hop chosen to propagate the data. Using the fuzzy C-means algorithm for clustering and the particle swarm optimization algorithm to optimize the initial center of the cluster, reference [71] proposed a clustering routing algorithm that ignores the fuzzy C-means and sensitivity to the center of the original cluster.

Table 1 summarizes the performance of various state-of-the-algorithms and the proposed method in terms of important metrics.

**Table 1:** Performance comparison of algorithms.

Reference	Target detection	Feature selection	Dataset	Machine learning technique	SDN	IoT	UDP
[51]	DoS, R2L, Probe, U2R	Information gain	NSL-KDD	Support vector machine	X	X	✓
[52]	DoS, R2L, Probe, U2R	Mutual information	NSL-KDD	Support vector machine	X	X	✓
[53]	DDoS	-	NSL-KDD99	Support vector machine	X	✓	X
[54]	DoS, R2L, Probe, U2R	Information gain	NSL-KDD99	XGBoot	X	X	✓
[55]	DoS, R2L, Probe, U2R	Regularized random forest	NSL-KDD99	Naïve Bayes	X	X	✓
[56]	Normal and attack	Stacked based feature selection	NSL-KDDCup99	Gated Recurrent Unit	X	X	✓
[57]	DDoS	-	GSB, IBRL	Random Forest	X	X	✓
[58]	DDoS	Principal component analysis	PDG, LUCE	Q-Learning	X	X	✓
[59]	DDoS	Information gain	KDD99	Online Naïve Bayes	X	✓	✓
[60]	DoS, R2L, Probe, U2R	Stacked based feature selection	KDD99	Random Forest	X	X	✓
[61]	Spike, drift, constant, noise	-	NSKL-DD	Linear Weighted Projection Regression	X	X	✓
[62]	Spike, drift, constant, noise	Information gain	Simulated data	Ensemble Learning	X	X	✓
[63]	Normal and attack	Chi-square information gain	KDD99	Passive Aggressive Algorithm	X	X	✓
[64]	DDoS	Mutual information	NAMOS, GSB	Stack Ensemble Learning	X	✓	✓
[65]	DoS	Information gain	KDD99	Leat-Square SVM	X	X	✓
[66]	DoS	Regularized random forest	UNSW-NB15	Random Forest	✓	X	✓
[67]	DoS	Mutual information	KDD99	Q-Learning	✓	X	X
[68]	DoS	-	Simulated data	Ensemble Learning	✓	X	✓
[74]	DoS	Information gain	NSL-KDD	Naïve Bayes	X	✓	✓
[75]	Spike, drift, constant, noise	-	KDD99	Support Vector Machine	X	X	✓
[76]	DDoS	Mutual information	KDD99	FCM	✓	✓	X
[77]	Spike, drift, constant, noise	-	NSL-KDD	Random Forest	X	X	✓
[78]	DoS, R2L, Probe, U2R	-	KDD99	ANN	X	✓	✓
[79]	DDoS	Regularized random forest	KDD99	SVM	X	✓	✓
[80]	DoS	-	GSB, PDG	Reinforcement learning	X	✓	X
[81]	Normal and attack	-	KDD99	Q-Learning	X	✓	X
Proposed	DDoS	Population diversity	GSB, LUCE, IBRL, PDG, NAMOS	FA, GSA	✓	✓	✓

### 2.1 Problem statement

In summary, the improved algorithm based on the traditional clustering routing protocol usually selects the cluster heads based on variables such as residual energy and distance, but it has the disadvantage of alternately selecting the clusters based on the probability and determine the number of key clusters spontaneously. The impact of cluster head position on node power consumption is often overlooked, despite the fact that some researchers try to delay the cluster head rotation as much as possible by setting a threshold.

Aiming at the above problems, in order to improve the efficiency of the optimization process for identifying an approximative global solution, a hybrid optimization algorithm that combines the global optimization powers of FA with the local optimization capabilities of BBOGSA is created. A distributed high-efficiency entropy energy-saving clustering routing system is created to speed up the rotation of the cluster heads. In order to show how well the hybrid optimization approach performs, it is compared to similar algorithms in terms of convergence accuracy, running time, and stability through experimental simulation and analysis. It is compared to competing techniques to show how the distributed high-efficiency entropy energy-saving clustering routing algorithm may lengthen network life and reduce data transmission energy usage.

## 3 System model

### 3.1 Network model

**Fig 1:**
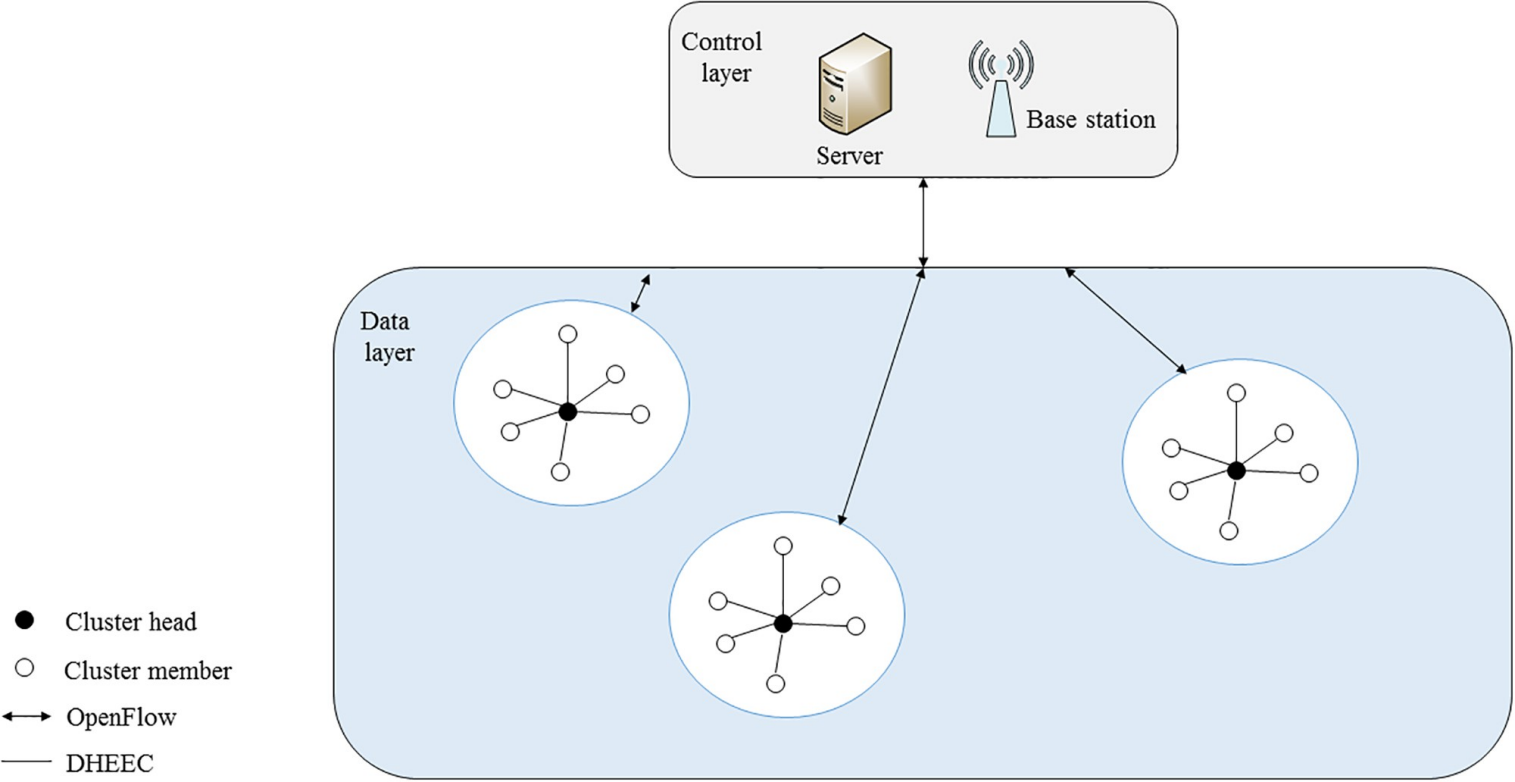
Proposed network model of SDWSN.

In the architecture of SDWSN, the base station is located at the control layer, including the controller module that calculates the route of the entire network. The sensor nodes are located in the data layer and realize the function of forwarding data according to the flow table [72–74]. According to the SDWSN architecture, the SDWSN model is shown in Fig 1, and the control layer has the following functions: to obtain information such as network topology, the remaining energy of sensor nodes, and location. Calculate the cluster head rotation table according to the energy, the optimal path to meet the demand, and load the DHEEC and cluster head rotation table into flow table information. The cluster head located in the data layer only completes receiving, merging, and forwarding data [75,76].

The network model is based on the following assumptions:

1. The base station is situated in the middle of the *M* × *M* area, and the sensor nodes *s*_*i*_ (*i* = 1,2, …, *N*) are dispersed at random [77–79];
2. Data communication between the cluster head and base station using the OpenFlow protocol [80–82];
3. Node software and hardware configurations are the same, each has a unique ID, and the location is fixed;
4. The links of the network are symmetrical;
5. The base station’s data transmission and calculation speeds are faster than those of the sensor nodes.

### 3.2 Energy consumption model

**Fig 2:**
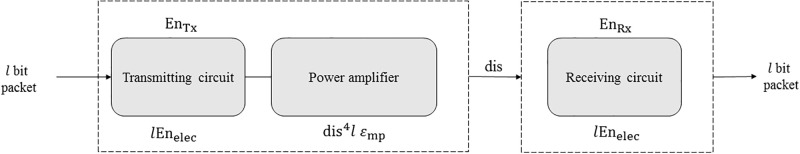
Energy consumption model.

The radio network energy consumption model is shown in Fig 2.

According to the energy consumption model, the energy En_Tx_ of transmitting a *l* bit packet is:

$$En_{\text{Tx}}=\left\{\begin{array}{l} l{\text{En}}_{\text{elec}}+{\text{dis}}^{2}l\varepsilon_{\text{fs}},\;\text{dis}<{\text{dis}}_{\text{break}}\\ l{\text{En}}_{\text{elec}}+{\text{dis}}^{4}l\varepsilon_{\text{mp}},\;\text{dis}\geq {\text{dis}}_{\text{break}} \end{array}\right.$$ (1)

Among them, En_elec_ is the unit energy consumption, *ε*_fs_ and *ε*_mp_ are the power amplification loss, dis is the distance [83,84], and dis_break_ is the threshold distance.

The energy *En*_Rx_ required to receive a *l* bit data packet is:

$$En_{\text{Rx}}=l{\text{En}}_{\text{elec}}$$ (2)

Therefore, the total energy consumed in one iteration is:

$$En_{\text{Round}}={\text{En}}_{\text{Tx}}+En_{\text{Rx}}=l_{\text{D}}\left(2N{\text{En}}_{\text{elec}}+N{\text{En}}_{\text{DA}}+k\varepsilon_{\text{mp}}{\text{dis}}_{\text{HB}}^{4}+k\varepsilon_{\text{fs}}{\text{dis}}_{\text{MH}}^{2}\right)$$ (3)

Among them, *k* is the number of clusters [85,86], dis_HB_ is the average distance between the cluster head and the base station, and dis_MH_ is the average distance between the cluster member node and the cluster head node:

$$\left\{\begin{array}{l} {\text{dis}}_{\text{HB}}=\frac{0.765}{2}M\\ {\text{dis}}_{\text{MH}}=\frac{M}{\sqrt{2\pi k}} \end{array}\right.$$ (4)

## 4 Hybrid optimization algorithm

### 4.1 Improved firefly algorithm

FA is an algorithm inspired by the flickering behavior of fireflies, which iterate their positions according to the brightness to find the optimal solution. The following rules are used:

1. Fireflies attract one-way according to the brightness;
2. The attraction of fireflies is proportional to the brightness;
3. Fireflies with low brightness are attracted to fireflies with high brightness;
4. The firefly with the highest brightness moves randomly.

**Definition 1**: The relative fluorescence brightness *I* of fireflies *i* and *j* is:

$$I=I_{0}e^{-\beta r_{ij}}$$ (5)

Among them, *I*_0_ is the maximum fluorescence brightness of fireflies, *β* is the light intensity absorption coefficient [87,88], *r*_*ij*_ is the Euclidean distance, and the calculation formula is:

$$r_{ij}=\|c_{i}-c_{j}\|=\sqrt{\sum_{w=1}^{D}{\left(c_{iw}-c_{jw}\right)}^{2}}$$ (6)

Among them, *c*_*i*_ and *c*_*j*_ represent the spatial positions of fireflies *i* and *j*, and *c*_*iw*_ and *c*_*jw*_ are the *w*-th dimension coordinates of fireflies *i* and *j*.

**Definition 2**: Mutual attraction *γ* between fireflies. *γ* corresponds to the relative fluorescence brightness between fireflies:

$$\gamma =\gamma_{0}e^{-\beta r_{ij}^{2}}$$ (7)

Among them, *γ*_0_ is the maximum attractiveness.

However, the convergence speed of FA is slow, and the global search lacks randomness, which may lead to convergence to a locally optimal solution [89,90]. The average distance between firefly individuals and ideal firefly individuals is used in this study to quantify population diversity and to suggest a location update technique based on population diversity.

**Definition 3**: Diversity index *β*^*n*^. Reflects the diversity of the *n-*th generation population:

$$\beta^{n}=\frac{1}{\left|s\right|}\sum_{i=1}^{\left|s\right|}\sqrt{\sum_{j=1}^{N}{\left(C_{ij}-{\bar{C}}_{\text{Y}}\right)}^{2}}$$ (8)

where *s* stands for population size, and ${\bar{C}}_{\text{Y}}$ denote the population’s mean center.

Further adjust the position update strategy of fireflies, as shown in formula (9):

$$\begin{array}{ll} c_{i}^{n+1} & =c_{i}^{n}+\gamma_{0}e^{-\beta r_{ij}^{2}}\left(c_{j}-c_{i}\right)+\alpha \times \text{rand}\\ & =c_{i}^{n}+\gamma_{0}e^{-\beta r_{ij}^{2}}\left(c_{j}-c_{i}\right)+\rho \left(c_{i}^{n}-C_{\text{best}}^{n}\right)+\alpha \times \text{rand} \end{array}$$ (9)

Among them, $c_{i}^{n}$ represents the spatial position of firefly *i*; *α* ∈ [0,1] represents the step factor; random parameter rand ∈ [0, 1]. $C_{\text{best}}^{n}$ represents the position of the brightest firefly individual in the current iteration n rounds [91–93]; *ρ* is a weight that changes according to *β*^*n*^ and the number of iterations, and its calculation formula is:

$$\rho =\left\{\begin{array}{l} -k\sigma ,\;\beta^{n}\leq \sigma \beta^{0}\\ 0,\beta >\sigma \beta^{0} \end{array}\right.$$ (10)

Among them, *β*^0^ is the initial population diversity index, k is the current iteration number, *σ* is a linear decreasing function, and its calculation formula is:

$$\sigma =\frac{\text{maxiterate}-k}{\text{maxiterate}}$$ (11)

Among them, maxiterate is the maximum number of iterations.

According to formula (11), the initial value of *σ* is 1, and when *k* = 1 in the first iteration, the value of *ρ* obtained by formula (10) is negative. Move in random directions away from the optimal solution, thus enabling a wider search [94]. Fireflies will do local random searches near the ideal solution to provide a more accurate local search as the number of iterations rises and the diversity index falls. In order to prevent the algorithm from converging to the local optimal solution, such a dynamic adjustment mechanism can balance the issues of global optimization in the early stages of the algorithm and local optimization in the later stages.

The fixed step size may make it difficult to reliably converge to the optimal point as the number of iterations rises since most fireflies would do local optimal searches in the vicinity of the optimal point. As a result, the step size factor is dynamically modified in the manner shown below:

$$\alpha =\alpha_{0}\sigma$$ (12)

where *α* is a constant value in FA. *α*_0_ represents the initial step size factor.

The specific steps of firefly algorithm are shown in Algorithm 1, and its steps are described as follows.

**Algorithm 1. Improved firefly algorithm.**

**Input**: population size *N*, initial step factor α, initial population diversity index *β*^0^, maximum number of iterations maxiterate

**Output**: optimal solution *C*_best_

 1: Determine objective function *f*(*x*)

 2: Create initial population *C*_0_

 3: Generate light intensity

 4: while *k* < maxiterate do

 5: Determine attraction *γ*

 6: for *i* ∈ [1, *N*] do

 7: for *j* ∈ [1, *N*] do

 8: Determine *σ*, *β*

 9: if *I*_*j*_ > *I*_*i*_ then

 10: Move firefly *i* towards *j*

 11: $c_{i}^{n+1}=c_{i}^{n}+\gamma_{0}e^{-\beta r_{ij}^{2}}\left(c_{j}-c_{i}\right)+\rho \left(c_{i}^{n}-C_{\text{best}}^{n}\right)+\alpha \times \text{rand}$

 12: end if

 13: Change attraction

 14: Search again and modify light intensity

 15: end for

 16: end for

 17: Determine the best solution *C*_best_

 18: end while

Algorithm 1 is explained below stepwise.

Step 1: Initialize parameters, population size *N*, initial step factor *α*, initial population diversity index *β*^0^, maximum iteration number maxiterate, etc.
Step 2: Construct the initial population *C*_0_, and calculate the brightness [95], sort according to the brightness, calculate the fitness *I* and the current global optimal solution *C*_best_ according to the maximum fluorescence brightness *I*_0_.
Step 3: Update the distance between fireflies according to formula (9), update the attraction between fireflies according to formula (7), and update the global optimal solution *C*_best_ at the same time.
Step 4: Add 1 to the number of iterations, and update the step factor according to formula (12).
Step 5: If the number of iterations reaches maxiterate, the algorithm stops and outputs the current global optimal solution *C*_best_, otherwise, execute steps 2 to 4 in a loop.

### 4.2 Gravity search and biogeography hybrid algorithm

BBO regards the solutions in the population as habitats, assigns a habitat suitability index (HSI) to each habitat to reflect the overall suitability, and updates the optimal solution through the population migration operation. Habitats are similar to firefly individual locations in FA [96], and the general rules used by BBO are as follows:

1. Brightness is proportional to the HSI value of the habitat;
2. Individuals in habitats with high HSI values migrate to habitats with low HSI values;
3. Habitats with low HSI values attract individuals from habitats with high HSI values;
4. Habitats face random variation among individuals.

**Definition 4**: Habitat *H*. Habitat provides a solution within the search space of a numerical optimization problem.

**Definition 5**: Habitat Suitability Index HSI. Reflect the overall suitability of the habitat.

**Definition 6**: Suitability index variable (SIV). The HSI is influenced by additional coefficients, such as rainfall, vegetation diversity, and temperature.

**Definition 7**: Emigration index *μ*. Controlling habitat migration, the probability of a species leaving a habitat is proportional to the number of species in the habitat.

**Definition 8**: Immigrant index *λ*. Controlling for habitat migration, the migration index is maximized when the habitat is free of species.

The BBO starts from a random population with population size *N* and dimension *D*, and each individual *H*_*ij*_ is generated according to formula (13).


$$H_{ij}=a_{j}+rand\times \left(b_{j}-a_{j}\right)$$ (13)

Among them, *i* = {1, 2, …, *N*}, *j* = {1, 2, … *D*}, *a*_*j*_ and *b*_*j*_ are the upper and lower bounds of the *j*th dimension of the solution vector respectively.

To calculate HSI, sort population individuals *H*_*k*_ in order from good to bad, and calculate emigration index *μ*_*k*_ and immigrant index *λ*_*k*_ according to formula (14).


$$\left\{\begin{array}{l} \mu_{k}=E\times S_{k}/S_{\text{max}}\\ \lambda_{k}=\left(1-S_{k}/S_{\text{max}}\right)I \end{array}\right.$$ (14)

Among them, *S*_*k*_ denotes the number of individuals in the current population, *S*_max_ represents the number of individuals in the largest population, *E* represents the maximum immigrant emigration rate, and *I* represents the maximum immigrant immigrant rate. The larger *μ*_*k*_ is, the smaller *λ*_*k*_ is, and the higher the HSI is. The smaller *μ*_*k*_ is, the larger *λ*_*k*_ is, and the lower the HSI. In each iteration, the better solution is propagated to the worse solution. The poorer solution has a high probability of learning from the better solution. Overall, as the number of iterations increases, all solutions approach the optimal solution.

Habitats with higher and lower HSIs are prone to mutation, while solutions in the middle are left untouched. The rate of variation is:

$$m_{i}=M\times \left[\frac{1-P_{i}}{P_{\text{max}}}\right]$$ (15)

Among them, *M* is the maximum mutation rate, *P*_*i*_ is the species probability, and *P*_max_ is the maximum species probability, where *P*_max_ = *N*.

Variation is caused by unexpected events that lead to changes in the number of species and HSI in the habitat, and the variation rate of species depends on the probability of species. When the number of species is large or small, the species probability is low and the variation rate is high; when the number of species is moderate, the species probability is high and the variation rate is low. The purpose of the mutation is to increase the diversity of the population so that the habitats with low HSI values are improved, the habitats with high HSI values are improved, and the habitats with high HSI values may change to low HSI values. The mutation is conducive to expanding the search space and jumping out of local optimum.

The gravitational force *F* between objects and the acceleration *a* of the individual are respectively:

$$\left\{\begin{array}{l} F=G\frac{M_{1}M_{2}}{R^{2}}\\ a=\frac{F}{M} \end{array}\right.$$ (16)

Among them, *M*_1_ and *M*_2_ are the mass of the object, *G* is the gravitational constant, *R* is the Euclidean distance, and *M* is the mass.

According to GSA, the gravitational force *f*_*ij*_ between population individuals *i* and *j* and the acceleration ai of individuals are respectively:

$$a_{i}=\frac{F_{ij}}{M_{ii}}$$ (17)

Among them, *M*_*aj*_ and *M*_*pi*_ represent the active gravitational mass of population individual *i* and the passive gravitational mass of population individual *j* respectively, and *M*_*ii*_ is the inertial mass of population individual *i*.

The inertial mass of population individuals is related to fitness. The larger the inertial mass, the greater the probability of producing an optimal solution. The mass *M*_*i*_(*t*) of the population at time *t* is calculated as follows:

$$\left\{\begin{array}{l} M_{ai}=M_{pi}=M_{ii}=M_{i},\;i=1,2,\ldots ,N\\ m_{i}\left(t\right)={\text{fit}}_{i}\left(t\right)-\text{worst}\left(t\right)/\text{best}\left(t\right)-\text{worst}\left(t\right)\\ M_{i}\left(t\right)=m_{i}\left(t\right)/\sum_{j=1}^{n}m_{j}\left(t\right) \end{array}\right.$$ (18)

Among them, fit_*i*_(*t*) is the fitness value, best(*t*) is the optimal solution, worst(*t*) is the worst solution, and the calculation formula is:

$$\left\{\begin{array}{l} \text{best}\left(t\right)=\underset{i\in \left\{1,2,\ldots ,N\right\}}{\text{max}}\;\;{\text{fit}}_{i}\left(t\right)\\ \text{worst}\left(t\right)=\underset{i\in \left\{1,2,\ldots ,N\right\}}{\text{min}}\;\;{\text{fit}}_{i}\left(t\right) \end{array}\right.$$

The solution set of the BBO algorithm is:

$$m_{ti}=\left[\left(I_{i1}+E_{i1}\right),\left(I_{i2}+E_{i2}\right),\ldots ,\left(I_{iD}+E_{iD}\right)\right]$$ (19)

BBO avoids falling into the local optimal solution and premature algorithm through migration and mutation operations, and improves the overall stability of the population; that is, it improves the value of HSI. According to HSI, the fitness function is created as follows:

$$\begin{array}{ll} {\text{fit}}_{i}\left(t\right) & =\text{HSI}={\text{SIV}}_{i}+N+\lambda_{k}-\mu_{k}\\ & ={\text{SIV}}_{i}+N+\left[\frac{1-S_{k}}{S_{\text{max}}}\right]I-\frac{ES_{k}}{S_{\text{max}}}\\ & ={\text{SIV}}_{i}+N+\left[\frac{1-S_{k}\left(I+E\right)}{S_{\text{max}}}\right]\\ & ={\text{SIV}}_{i}+N+\left[\frac{1-S_{k}M_{t}}{S_{\text{max}}}\right] \end{array}$$ (20)

Update formula (18) to get:

$$m_{i}\left(t\right)=\frac{{\text{S}}_{\text{max}}\left[{\text{SIV}}_{i}+N+I-S_{k}M_{t}-\text{worst}\left(\text{t}\right)\right]}{{\text{S}}_{\text{max}}\left[\text{best}\left(\text{t}\right)-\text{worst}\left(\text{t}\right)\right]}$$ (21)

Update the speed and position of the individual according to formula (17):

$$v_{i}^{d}\left(t+1\right)={\text{rand}}_{i}\times v_{i}^{d}\left(t\right)+a_{i}^{d}\left(t\right)$$ (22)

$$x_{i}^{d}\left(t+1\right)=v_{i}^{d}\left(t\right)+v_{i}^{d}\left(t+1\right)$$ (23)

The pseudocode of BBOGSA is shown in Algorithm 2, and its steps are described as follows.

**Algorithm 2. BBOGSA algorithm.**

**Input**: population size *N*, dimension *D*, maximum immigrant emigration rate *E*, maximum immigrant immigrant rate *I*, maximum mutation rate *M*, maximum number of iterations *r*_max_

**Output**: Optimal solution *G*_best_

 1: Determine objective function *f*(*x*) = *C*_best_

 2: Generate initial population *C*_0_

 3: while *k* < *r*_max_ do

 4: Determine HIS

 5: for *i* ∈ [1, *N*] do

 6: for *j* ∈ [1, *N*] do

 7: Calculate *μ*_*k*_, *λ*_*k*_, *M*

 8: if *M*_*j*_ > *M*_*i*_

 9: Move *i*th habitat toward *j*th

 10: $\text{HSI}={\text{SIV}}_{i}+\left[\frac{I-S_{k}M_{t}}{S_{\text{max}}}\right]$

 11: end if

 12: Change HIS

 13: Search and modify again gravitational

 14: end for

 15: end for

 16: Determine the optimal solution *G*_best_ by sorting HIS

 17: end while

The steps of the Algorithm 2 are explained below.

Step 1: Set parameters such as the initial population size *N*, dimension *D*, maximum immigrant emigration rate *E*, maximum immigrant immigration rate *I*, maximum mutation rate *M*, and maximum number of iterations *r*_max_.
Step 2: Construct the initial population *C*_0_, calculate the suitability index HSI, sort and update the current global optimal solution *G*_best_.
Step 3: Calculate the migration index *μ*_*k*_ and the immigration index *λ*_*k*_ of the habitat according to the formula (14), and perform the migration operation, and perform the mutation operation according to the formula (15).
Step 4: Calculate the inertial mass of each population according to formula (21), and update the individual position according to formula (23).
Step 5: If the number of iterations reaches *r*_max_, the algorithm stops and outputs *G*_best_, otherwise, execute steps 2 to 4 in a loop.

### 4.3 Hybrid optimization algorithm design

FA tends to converge to the local optimal solution in the final iteration, which makes it unable to perform a proper local search. The local search ability of BBOGSA makes up for the defect of FA. Therefore, FGB is designed to combine the global search ability of FA and the local search ability of BBOGSA to improve the performance of the optimization process for finding approximate global solutions.

FGB has two phases. In the first stage, the improved FA performs preliminary optimization to search the design search space by performing a limited number of iterations, and the optimal solution found in this stage is *C*_*best*_. In the second stage, a more refined search of *C*_best_ is performed using BBOGSA algorithm for a limited number of iterations.

The initial population constructed by the improved FA is randomly distributed in the whole space, while in BBOGSA, *C*_best_ is directly transformed into the optimal initial population.

**Fig 3:**
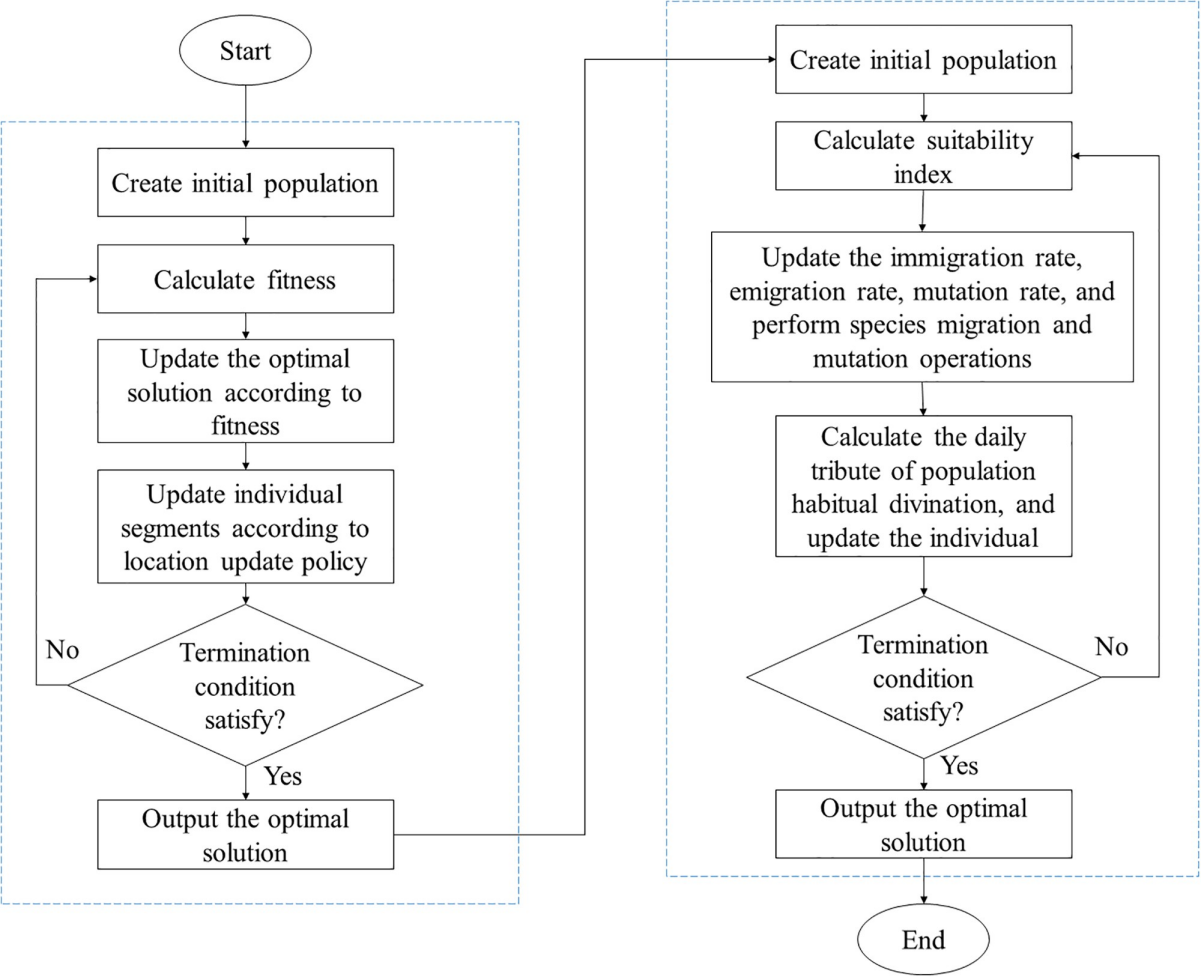
Flowchart of FGB mechanism.

The FGB process is shown in Fig 3.

The pseudocode of FGB is shown in Algorithm 3.

**Algorithm 3. FGB algorithm.**

**Input**: *L*, *N*, *α*, *β*^0^, maxiterate, *D*, *E*, *I*, *r*_max_

**Output**: Optimal solution *G*_best_

 1: Determine objective function *f*(*x*)

 2: Create initial population *C*_0_

 3: Generate light intensity

 4: while *k* < maxiterate do

 5: Determine *γ*

 6: for *i* ∈ [1, *N*] do

 7: for *j* ∈ [1, *N*] do

 8: Determine *β*, *σ*

 9: if *I*_*j*_ > *I*_*i*_ then

 10: Move *i*th firefly towards *j*th

 11: $c_{i}^{n+1}=c_{i}^{n}+\gamma_{0}e^{-\beta r_{ij}^{2}}\times \left(c_{j}-c_{i}\right)+\rho \times \left(c_{i}^{n}-c_{\text{best}}^{n}\right)+\alpha \times \text{rand}$

 12: end if

 13: Change *γ*

 14: Determine light intensity again and modify it

 15: end for

 16: end for

 17: Determine the optimal solution *C*_best_

 18: end while

 19: Determine objective function *f*(*x*) = *C*_best_

 20: Create initial population *C*_0_

 21: while *k* < *r*_max_ do

 22: Determine HIS

 23: for *i* ∈ [1, *L*] do

 24: for *j* ∈ [1, *L*] do

 25: Determine *μ*_*k*_, *λ*_*k*_, *M*

 26: if *M*_*j*_ > *M*_*i*_

 27: Move *i*th habitat towards *j*th

 28: Calculate $\text{HSI}={\text{SIV}}_{i}+N+\left[\frac{I-S_{k}M_{t}}{S_{\text{max}}}\right]$

 29: end if

 30: Change the variable HIS

 31: Modify gravitational by searching again

 32: end for

 33: end for

 34: Determine the optimal solution *G*_best_ by sorting HIS

 35: end while

## 5 High entropy energy saving clustering routing algorithm

**Fig 4:**
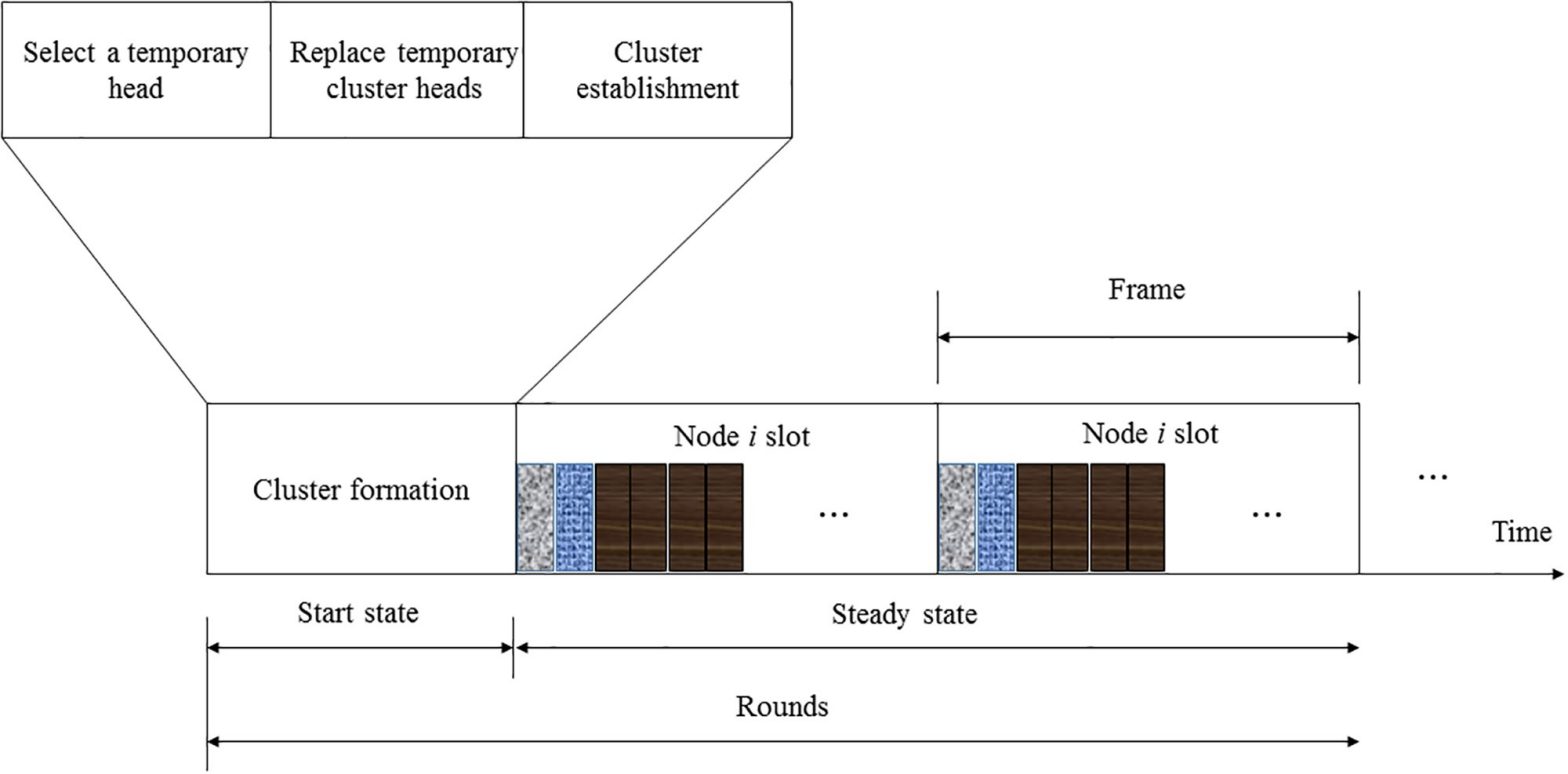
Running time sequence of DHEEC.

The running time sequence of DHEEC is shown in Fig 4, with rounds as the execution cycle, including the start-up state and steady state. Before the first round of iteration, the base station sends flow table information and forwards it to all sensor nodes, while at the same time indicating the start. In the initial stage of a startup, SDWSN has two important functions: (1) selecting the cluster head; (2) establishing a cluster. In (1), the temporary cluster head is selected based on the threshold calculation formula, and the node information is sent to the base station through the cluster head. Then, based on the FGB, the temporary cluster head is exchanged with the members of the cluster, so that the node with the highest energy becomes the cluster head. The cluster head sends a message to the adjacent nodes; the common node selects the cluster head to join the cluster and becomes a member node in the cluster; and the cluster is formally established. The SDWSN enters the stable stage and transmits data according to inter-cluster routing.

In DHEEC, entropy is used to describe the probability of a node predicting to become a cluster head, the information uncertainty of the node’s initial energy and residual energy, the smaller the entropy, the greater the residual energy of the node. Each sensor node automatically completes its own entropy selection by obtaining the initial energy and remaining energy levels.

### 5.1 Threshold formula

Based on the assumption of the system model, the initial energy of the sensor node is randomly distributed in the interval [En_0_, (1 + *a*_max_)En_0_], so the sensor node *s*_*i*_(*i* = 1, 2,…, *N*) has (1 + *a*_*i*_)En_0_ initial energy, where *a*_*i*_ ∈ [0, *a*_max_]. The initial total energy En_total_ of the network is shown in formula (24):

$${\text{En}}_{\text{total}}=\sum_{i=1}^{N}\left(1+a_{i}\right){\text{En}}_{0}={\text{En}}_{0}\left[N+\sum_{i=1}^{N}a_{i}\right]={\text{En}}_{0}\left(N+A\right)$$ (24)

**Definition 14**: The relative energy entropy *H*(En_*i*_) of the node is:

$$H\left({\text{En}}_{i}\right)=-\sum_{i=1}^{n}k\times {\text{En}}_{i}\left(r\right)\text{ln}\;\bar{E}\left(r\right)$$ (25)

Among them, En_*i*_(*r*) is the remaining energy of the sensor node *s*_*i*_ in *k* rounds, and $\bar{E}\left(r\right)$ represents the average energy of the r round network:

$$\bar{E}\left(r\right)=\frac{1}{N}\sum_{i=1}^{N}{\text{En}}_{i}\left(r\right)$$ (26)

The relative energy entropy consists of three parts: the predictable number of cluster heads, the remaining energy, and the average energy, and oscillates around in the direction of the two relative energies so that a single node can automatically manage its entropy. In each round of selecting a temporary cluster head, each node evaluates the probability of becoming a cluster head and extracts a random number between 0 and 1 as the conditional probability. If it is less than the conditional probability, the node will choose itself as a temporary cluster head.

The formula for cluster head selection threshold is:

$$T\left(n\right)=\left\{\begin{array}{l} H\left({\text{En}}_{i}\right)/\left[1-H\left({\text{En}}_{i}\right)\right]\left[r\text{mod}\left[\frac{1}{H\left({\text{En}}_{i}\right)}\right]\right],\;s_{i}\in C\\ 0,\;\text{other} \end{array}\right.$$ (27)

Among them, *C* represents the node group that has not become the cluster head, and participates in the selection of the *r*-th round of cluster head according to the threshold *T*(*n*). In the last round of algorithm iteration, if si does not become the cluster head, then *s*_*i*_ ∈ *C*. In this way, the conditional operation based on the relative energy entropy of node energy and initial energy is more suitable for energy update in SDWSN.

### 5.2 Cluster head election using FGB

FGB is used to identify the relative energy entropy in (23), using the following rules:

1. Fireflies attract one-way according to brightness, that is, node energy;
2. The attraction of fireflies is proportional to the node energy;
3. Fireflies with low energy are attracted to fireflies with high energy;
4. The firefly with the highest energy moves randomly.

In DHEEC, fireflies with low entropy, high attractiveness, and good location will attract a large number of fireflies and the attractiveness of fireflies can be predicted by the objective function, namely relative energy entropy. If the attractive forces are balanced, the fireflies will move arbitrarily.

In the algorithm, FGB is used for cluster head selection and is divided into two stages. In the first stage, the improved FA performs preliminary optimization, and the optimal solution found in this stage is *C*_best_;

In the second stage, a more refined search of *C*_best_ was performed using BBOGSA to find the optimal solution of is *G*_best_. The optimal solution is the node with the largest energy in the current iteration times.

### 5.3 Inter-cluster routing

**Fig 5:**
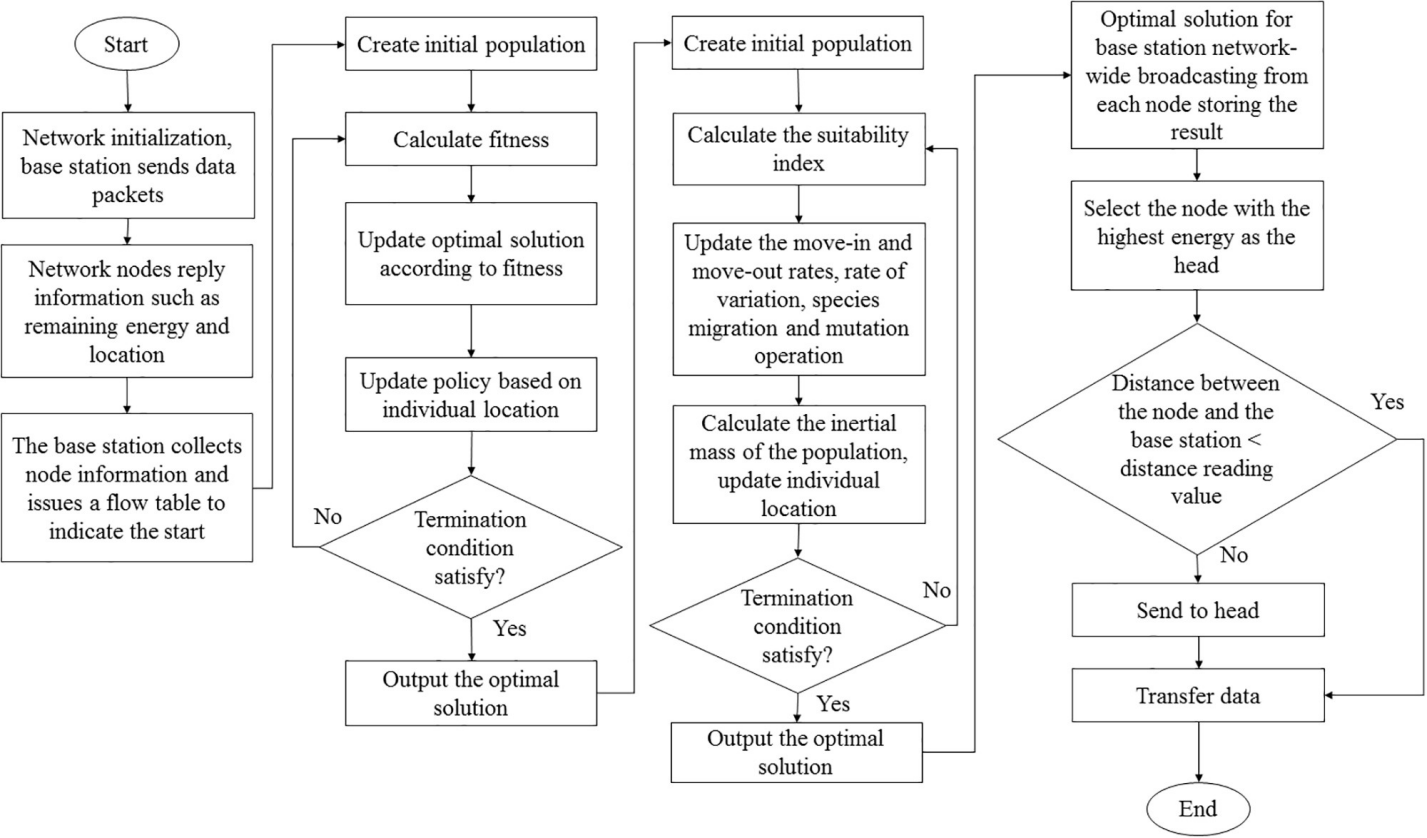
Flowchart of the proposed DHEEC algorithm.

Sensor nodes send node location information, remaining energy information, and topology information to the base station. The node data transmission mode is shown in Table 2, where d is the distance between the node and the base station and ‘dis’ is the distance threshold, that is, the distance from the node to the cluster head. The base station calculates the network route and controls the sensor nodes. This approach reduces energy consumption and computational overhead. The flow chart of the DHEEC algorithm is shown in Fig 5.

**Table 2:** Node data transmission method.

Data transfer method	Condition
Direct	*d* < dis
Cluster head	*d* ≥ dis

## 6 Simulation results

The software used in this section is MATLAB 2018a. First, verify the convergence performance of FGB by comparing the performance of PSOGWO [22], HFPSO [17] and FGB on different test functions; Secondly, by comparing the network lifetime and data transmission volume of IFCEER [15], DEEC-FA [20] and DHEEC, the network performance of DHEEC is verified. PSO is a swarm-based meta-heuristic optimization technique that moves particles in a designated exploration area in an attempt to find the optimum solution to a given issue. Conversely, Grey Wolf Optimization (GWO) is a meta-heuristic optimization approach that draws inspiration from wolves. This article aims to enhance the GWO’s advancement by hybridizing it with a PSO approach. A metaheuristic optimization approach called Hybrid Firefly and Particle Swarm Optimization (HFPSO) combines the best aspects of particle swarm and firefly optimization. By examining the prior global best fitness values, HFPSO attempts to accurately establish when the local search process began.

### 6.1 Standard function test experiment

The standard functions used for testing are shown in Table 3, and *f*_1_ is a unimodal function to examine the convergence speed and precision of the optimization algorithm. *f*_2_, *f*_3_, and *f*_14_ are multimodal functions, and examine the global search ability of the optimization algorithm. *f*_5_ is a discontinuous step function to examine the effectiveness of the optimization algorithm. The problem dimension is 30, and their optimal values are all 0.

**Table 3:** Standard functions to test.

Function name	Expression	Range
Sphere	$f_{1}\left(x\right)=\sum_{i=1}^{n}x_{i}^{2}$	[-100, 100]
Schwefel	$f_{2}\left(x\right)=\sum_{i=1}^{n}\left|x_{i}\right|+\prod_{i=1}^{n}\left|x_{i}\right|$	[-10, 10]
Rastrigin	$f_{3}\left(x\right)=\sum_{i=1}^{n}\left[x_{i}^{2}-10\;\text{cos}\left(2\pi x_{i}\right)+10\right]$	[-5.12, 5.12]
	$\begin{array}{l} f_{4}\left(x\right)\\ =-20\;e^{-0.2\sqrt{\frac{1}{n}\sum_{i=1}^{n}x_{i}^{2}}}\\ -e^{\left[\frac{1}{n}\sum_{i=1}^{n}\;\text{cos}\left(2\pi x_{i}\right)\right]+20+e} \end{array}$	[-32, 32]
Step	$f_{5}\left(x\right)=\sum_{i=1}^{n}{\left(x_{i}+0.5\right)}^{2}$	[-100, 100]

#### 6.1.1 Parameter settings

Some parameters of the test standard function experiment are set as follows: *N* = 30, *α* = 0.2, *β*^0^ = 1, *D* = 3, *E* = 1, *I* = 1.

**Fig 6:**
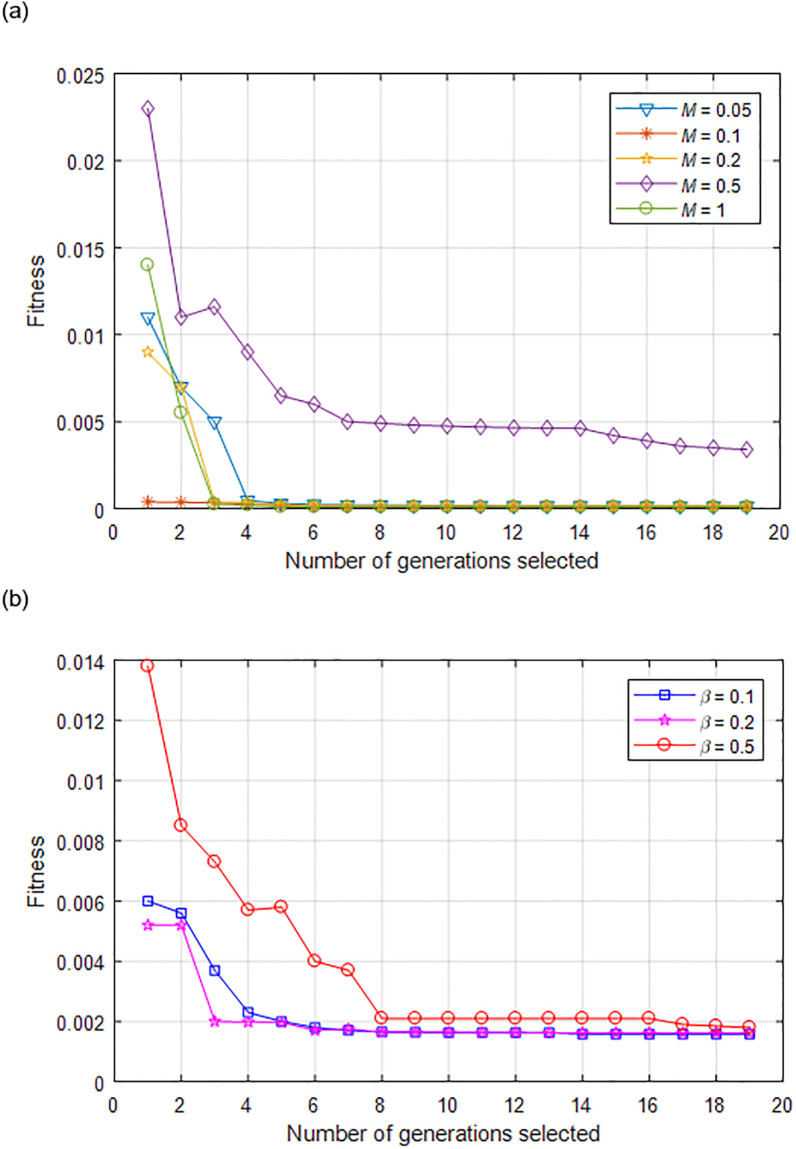
Changes in the fitness of FGB under different maximum mutation rates and population diversity indices. (a) Variation with *M*; (b) Variation with *β*.

The fitness changes of FGB under different maximum mutation rates and population diversity indices are shown in Fig 6. Fig 6(a) shows the fitness change of FGB when the maximum number of iterations is 20 rounds and the maximum mutation rate *M* is 0.05, 0.1, 0.2, 0.5 and 1 respectively. The mutation rate increases the diversity of individuals in the population, and a moderate mutation rate promotes the algorithm to quickly converge to the optimal value. Here, the maximum mutation rate is 0.1. Fig 6(b) shows the fitness changes of FGB when the population diversity index β is 0.1, 0.2 and 0.5 respectively. When the population diversity is moderate, the species probability is higher and the algorithm is more stable. At this time, the population diversity index is taken as 0.2.

#### 6.1.2 Results

The results of the three algorithms under the five standard functions are compared in Table 4. It can be seen from the results that FGB is slightly worse than PSOGWO on *f*_1_, but the convergence speed is faster than PSOGWO. The performance of FGB on *f*_2_ shows that its search solution range is large. FGB performs well on *f*_3_, *f*_4_ and *f*_5_, which shows its practicability, convergence speed and search ability are stronger than the other two algorithms.

**Table 4:** Comparison of results of algorithms under five standard functions.

Function	FGB		PSOGWO		HFPSO	
	Optimal value	Running time (s)	Optimal value	Running time (s)	Optimal value	Running time (s)
*f*_1_	9.0824×10^−17^	1.94	1.4335×10^−26^	2.21	4.7795×10^−9^	2.39
*f*_2_	3.715×10^−8^	1.97	9.465×10^−9^	2.05	5.6329×10^−5^	2.30
*f*_3_	20.8941	1.91	23.6247×10^−7^	1.98	36.813×10^−5^	2.11
*f*_4_	7.8769×10^−9^	2.27	374.867	1.85	2.8522×10^−5^	1.86
*f*_5_	0	2.08		1.19	0	1.47

### 6.2 Network simulation experiment

#### 6.2.1 Simulation parameter settings

Some parameters of the network simulation experiment are shown in Table 5.

**Table 5:** Network simulation parameters.

Parameter	Value
Network area	100 m×100 m, 250 m × 250 m
Number of sensor nodes	100 ~ 300
Base station location	(50, 50)
Transmission packet	4000 bits
Packet rate	4 packets/s
Number of nodes per cluster	6
Max battery level of node	3 J
*E*_0_	0.5 J
*E*_elec_	50 nJ/bit
*ε*_fs_	10 nJ/bit.m^2^
*ε*_mp_	0.0013 pJ/bit.m^4^
*E*_DA_	5 nJ/bit.signal

#### 6.2.2 Network life cycle

The energy of network nodes decreases as the number of iterations of the algorithm increases, and is exhausted after a certain number of iterations, resulting in the death of the node. The number of dead nodes in the network represents the life cycle of the network.

**Fig 7:**
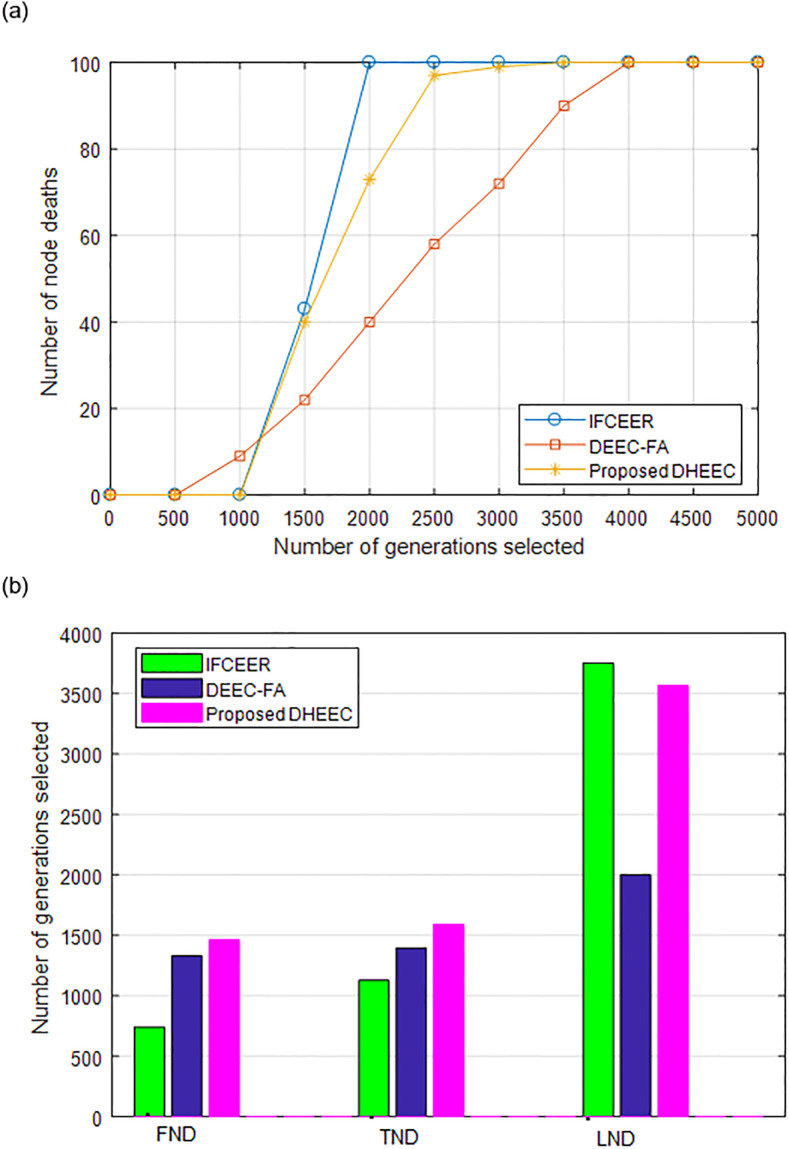
Comparison of network node life cycle of algorithms. (a) Number of node deaths. (b) Node death time.

The comparison of the network node life cycle of IFCEER, DEEC-FA and DHEEC is shown in Fig 7, which shows the impact of the three algorithms of IFCEER, DEEC-FA and DHEEC on the network life cycle. Fig 7(a) shows the changes in the number of dead nodes in the three algorithms of IFCEER, DEEC-FA and DHEEC when the maximum number of iterations is 5000 rounds. It can be seen that the number of death rounds of all nodes of the three algorithms of IFCEER, DEEC-FA and DHEEC is 2000, 3754 and 3568 respectively. The first node of the network dies, and the information collected by the base station is no longer comprehensive. With the operation of the algorithm, the number of dead nodes increases, and a small number of nodes that survive in the later stage have low remaining energy and lose communication capabilities. Therefore, in order to measure the life cycle of the network more comprehensively, Fig 7(b) compares the number of rounds of the first node death (first node died, FND), 10% node died rounds (10% node died, TND) and all nodes died rounds (last node died, LND). It can be seen that IFCEER only allows high-energy nodes to participate in the election of cluster heads, resulting in very close FND and LND. The FND of DEEC-FA is much smaller than the other two algorithms, and the network performance drops seriously in the early stage of the algorithm. Considering the balance of the overall energy consumption of the network, and defining TND as the network life cycle, the TND of DHEEC is about 41.05% higher than IFCEER, and about 13.89% higher than that of DEEC-FA.

#### 6.2.3 Network data transfer volume

During the operation of the algorithm, the surviving member nodes transmit the collected data to the cluster head, and then the cluster head transmits it to the base station. After the transmission of all nodes is completed, the base station counts the number of data packets received in this round. Therefore, the total number of data packets received by the base station is used to evaluate the energy utilization rate. The more data packets received by the base station, the more balanced the energy distribution will be.

**Fig 8:**
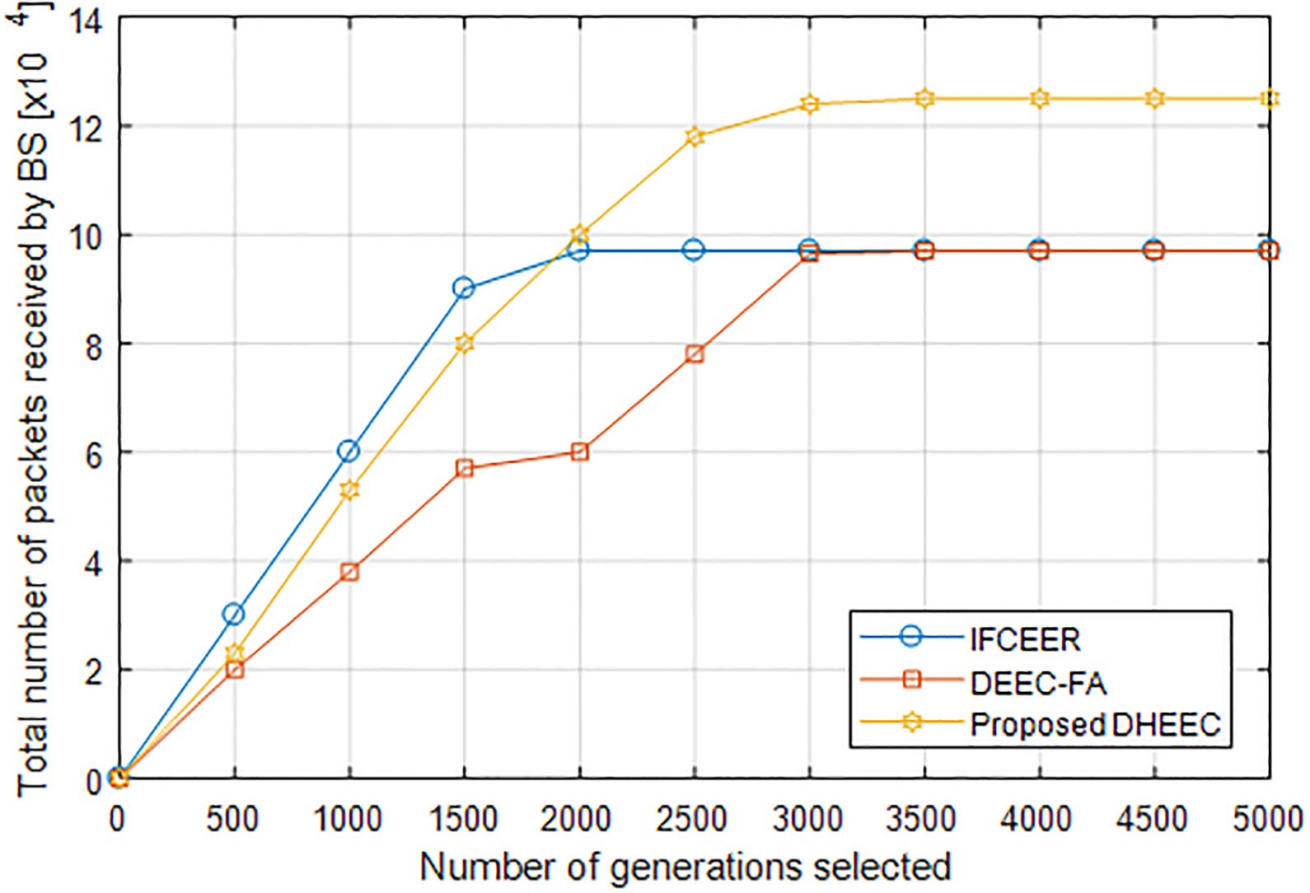
The total number of packets received by the base station.

The total number of data packets received by the base station is shown in Fig 8, which shows the change of the total number of data packets received by the base station after 5,000 iterations of the three algorithms of IFCEER, DEEC-FA and DHEEC. It can be concluded that the energy utilization rate of DHEEC is about 31.58% higher than that of IFCEER and about 31.06% higher than that of DEEC-FA.

**Fig 9:**
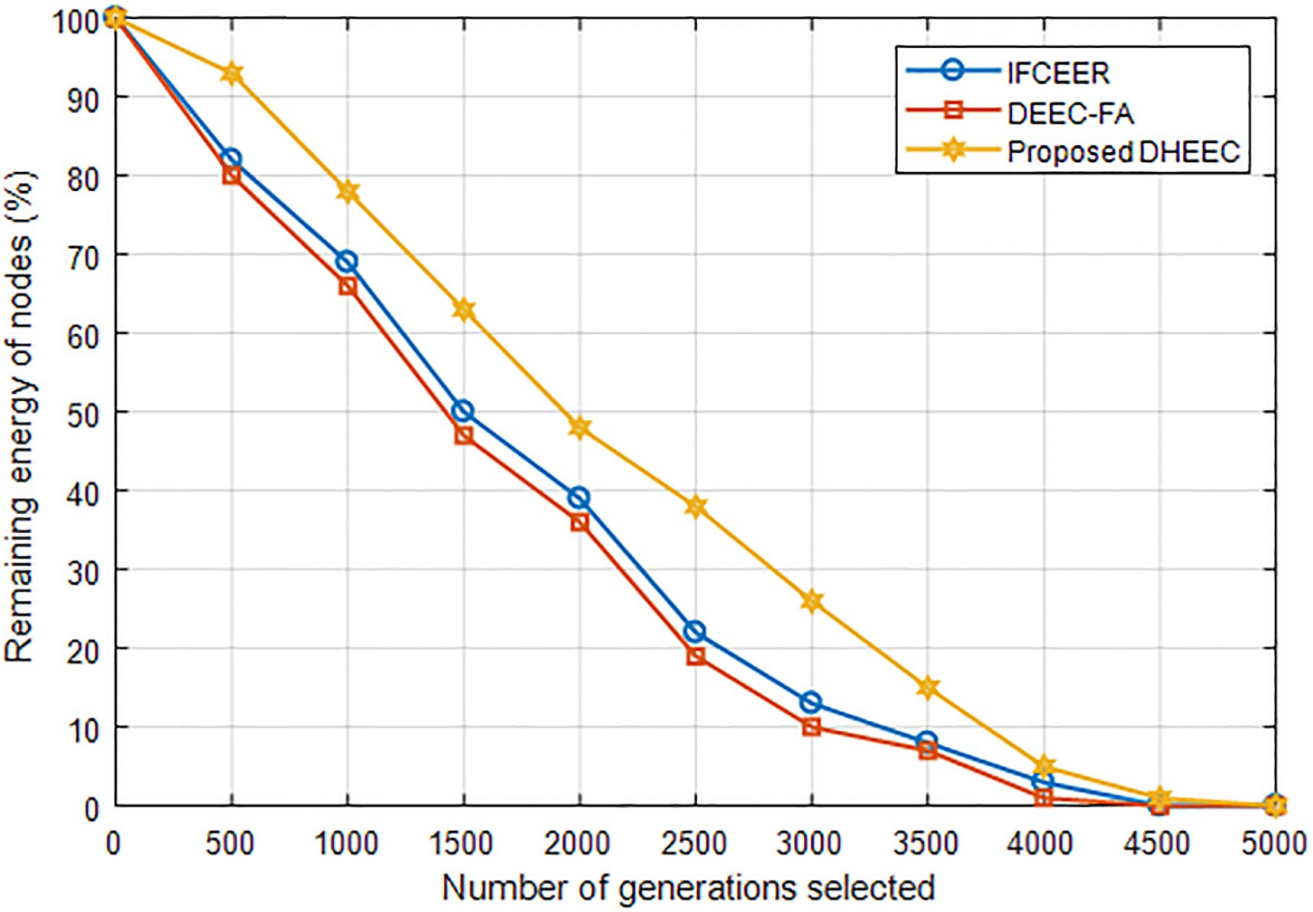
Comparison of average remaining energy of nodes of the algorithms.

Fig 9 compared the average remaining energy of nodes of the algorithms. As can be seen that the average remaining energy of the proposed algorithm is higher than the existing algorithms which makes it superior and has longer lifetime.

### 6.3 Statistical evaluation

**Fig 10:**
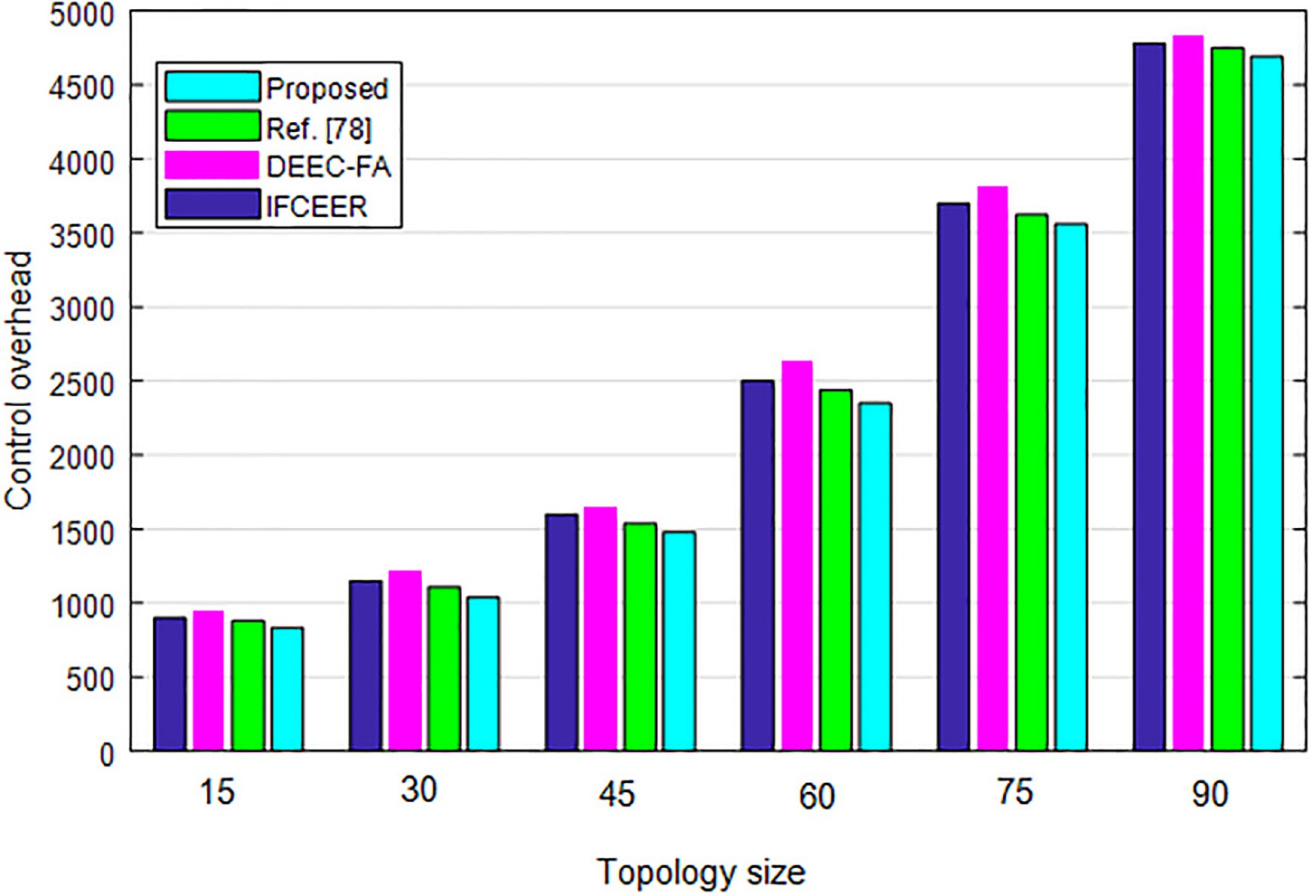
Control overhead comparison of algorithms under different topology size.

Fig 10 compares the control overhead of algorithms under different topology size. As can be seen from the results, the control overhead of the algorithms increases with increasing topology size. However, the overhead of the proposed method is lower than existing algorithms which validates its effectiveness.

**Fig 11:**
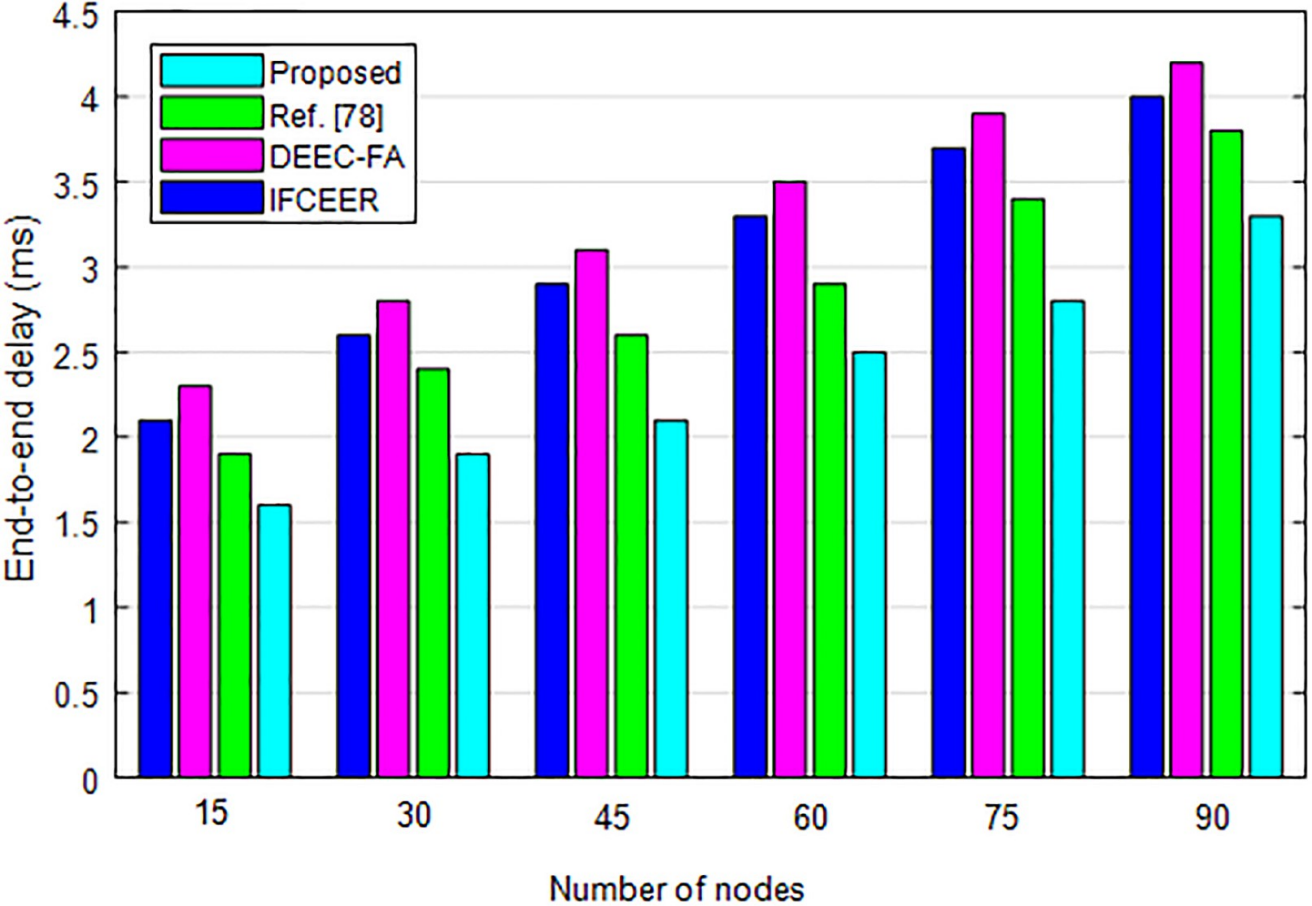
End-to-end delay comparison of algorithms under different number of nodes.

Fig 11 compares the end-to-end delay of the algorithms under increasing number of nodes. It can be seen from Fig 11, the delay increases with increasing number of nodes. However, the proposed method has lower delay than existing algorithms, which further validates its superior performance.

### 6.4 Scalability and performance in larger networks

**Fig 12:**
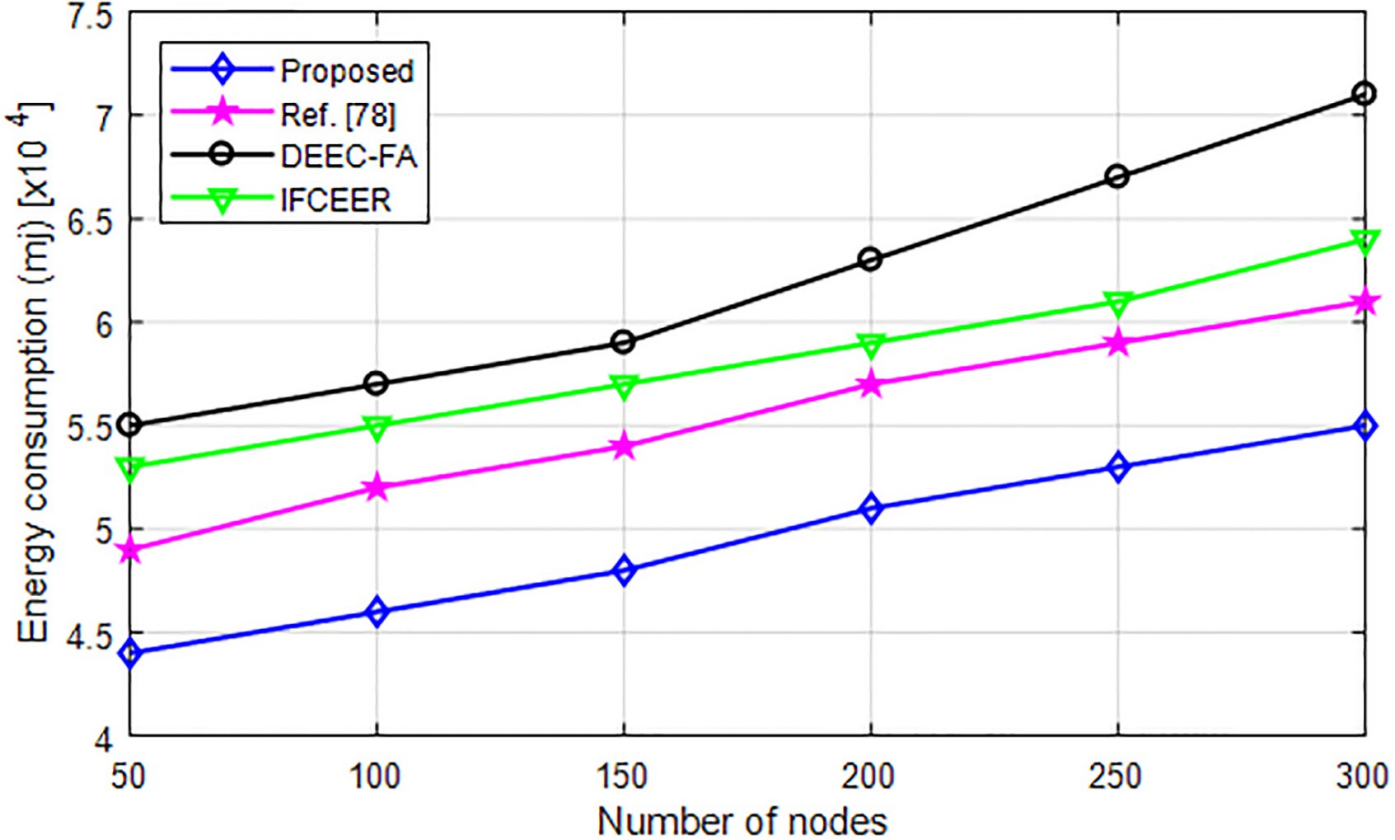
Scalability evaluation under large network.

Fig 12 evaluates the energy consumption of the network with dimension of 250 m×250 m and the number of nodes is 300. As can be seen from Fig 12, the energy consumption of the algorithms increases with increasing number of nodes. However, the proposed method consumes less energy as compared with existing algorithms which makes is energy-efficient.

### 6.5 Energy efficiency analysis under different network scenario

**Fig 13:**
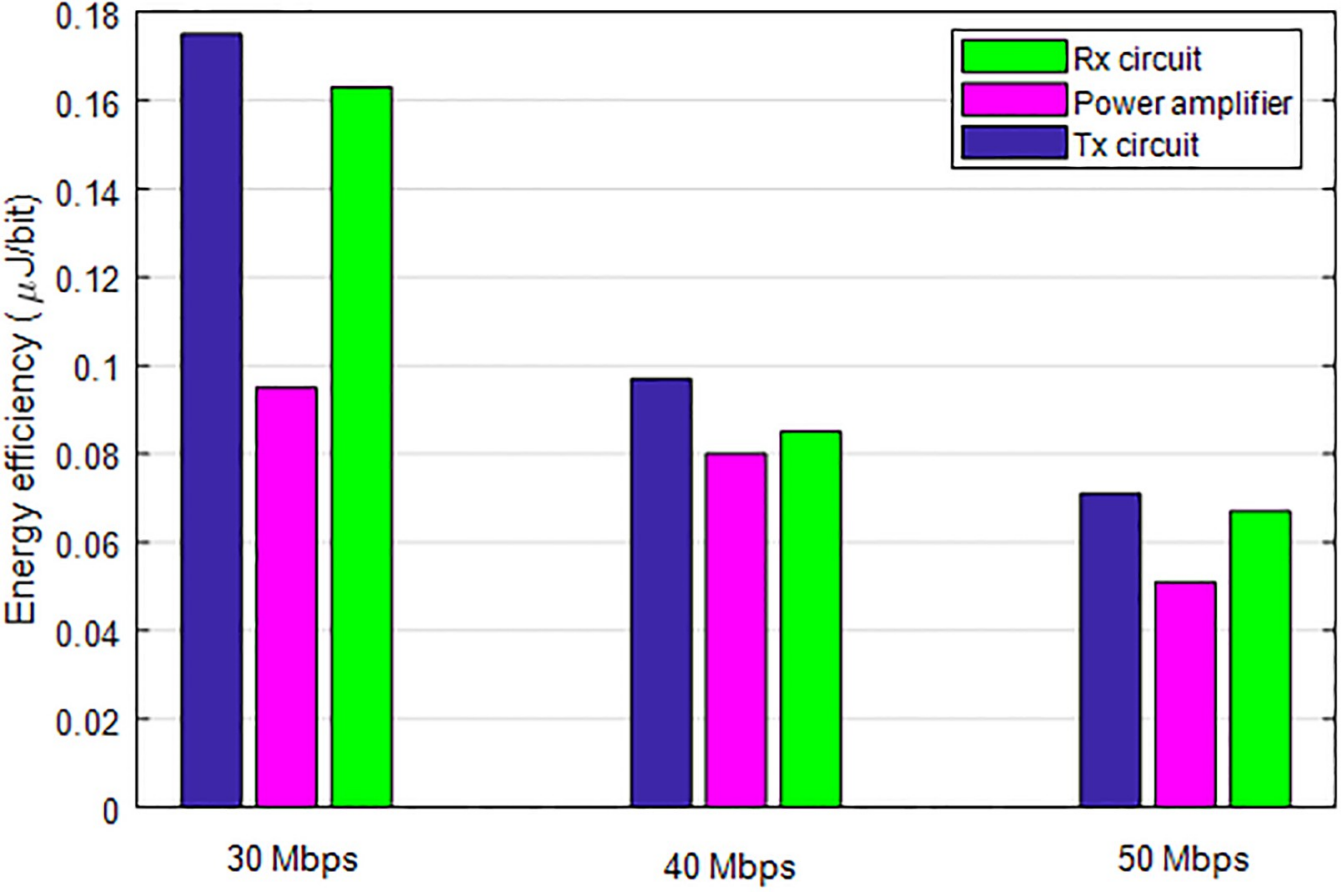
Energy efficiency analysis of each component.

Fig 13 evaluates the energy efficiency of the algorithms under different network scenario. As can be seen from Fig 13, the energy efficiency of each component reduces with increasing data rate. However, its within the required range of operation.

### 6.6 Impact on running time and computational resources

**Fig 14:**
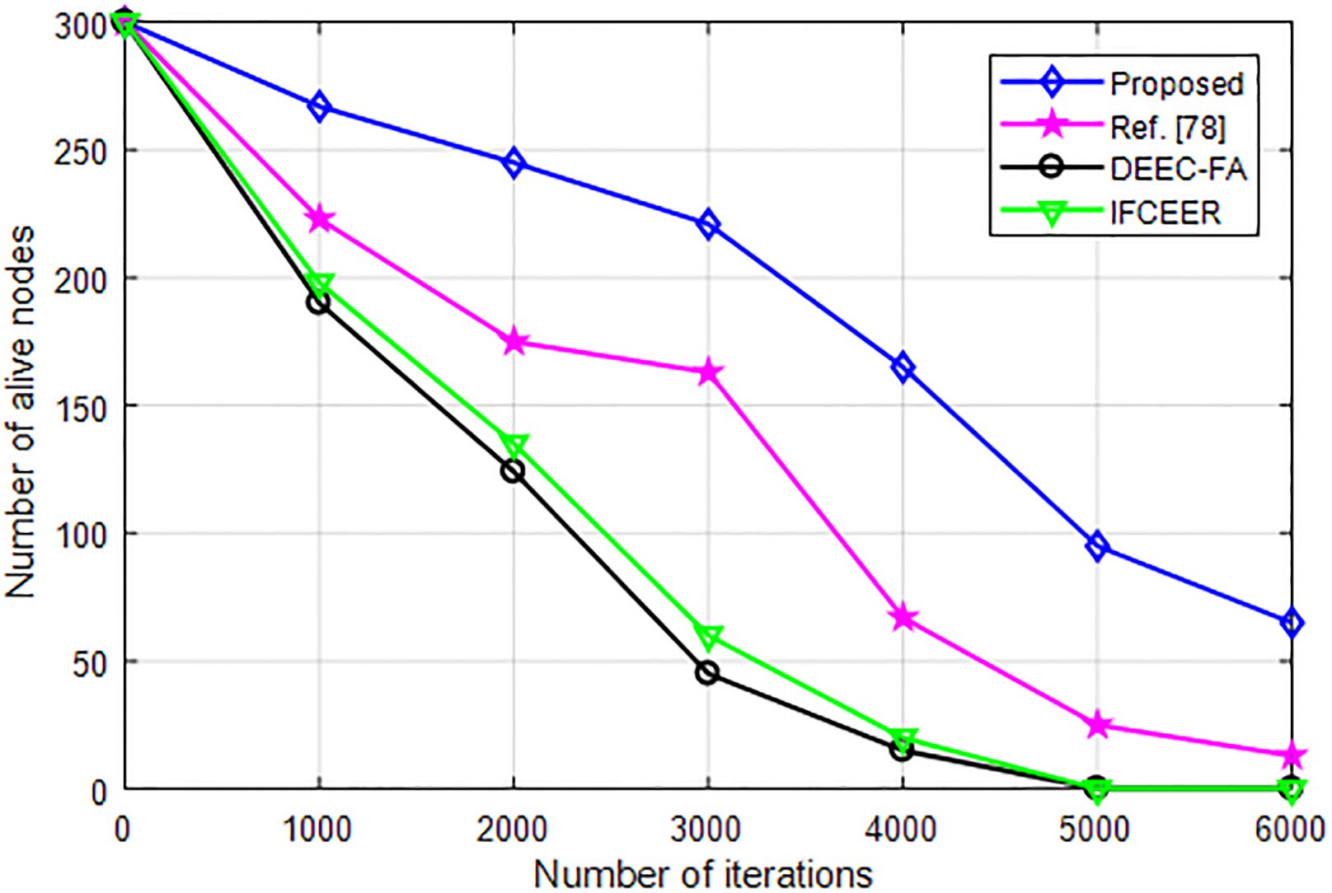
Network running time comparison of algorithms.

Fig 14 compares the running time of network of algorithms under increasing number of iterations. As can be seen from Fig 14, the network lifetime of the algorithms decreases with increasing number of iterations. However, the running time of the proposed algorithm is longer than existing methods which makes is reliable and require less computational resources.

### 6.7 Robustness to network dynamics

**Fig 15:**
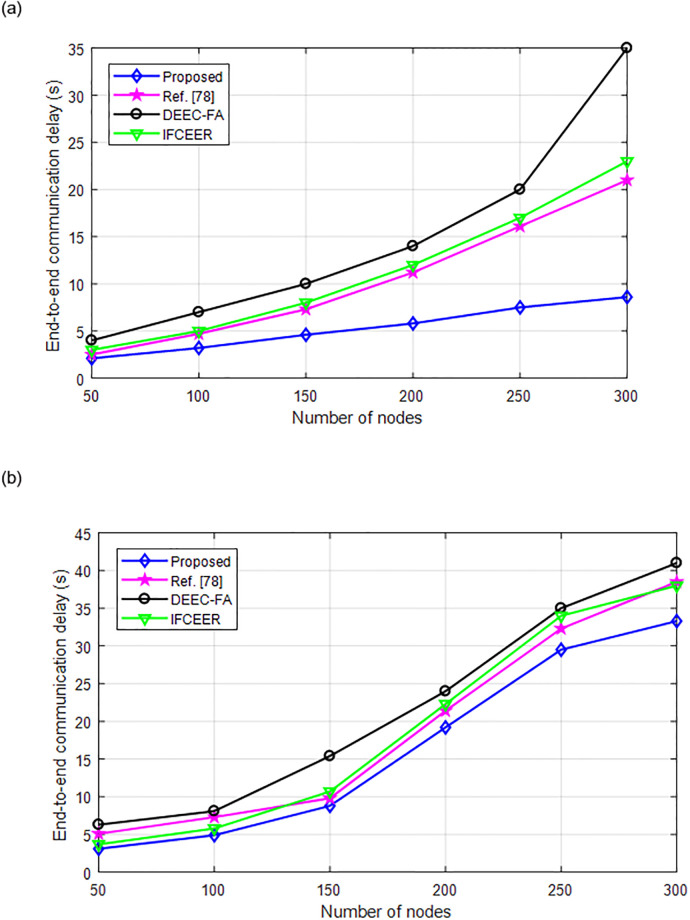
Communication delay evaluation at different node mobility. (a) 1.8 m/s. (b) 5.5 m/s.

Fig 15 compares the robustness of the algorithms when the mobile nodes move at different speed. As can be seen from Fig 15a, when the number of nodes increases, the communication delay increases. The communication delay of the proposed algorithm is lower than existing methods. From Fig 15b, the mobile node speed is 5.5 m/s and as the number of nodes increases, the communication delay at this speed is larger than at speed of 1.8 m/s. In both scenarios, the proposed method has lower communication delay which proved its effectiveness. This proves that, the proposed method is robust to dynamic network environment and with varying data traffic.

### 6.8 Convergence of algorithm

**Fig 16:**
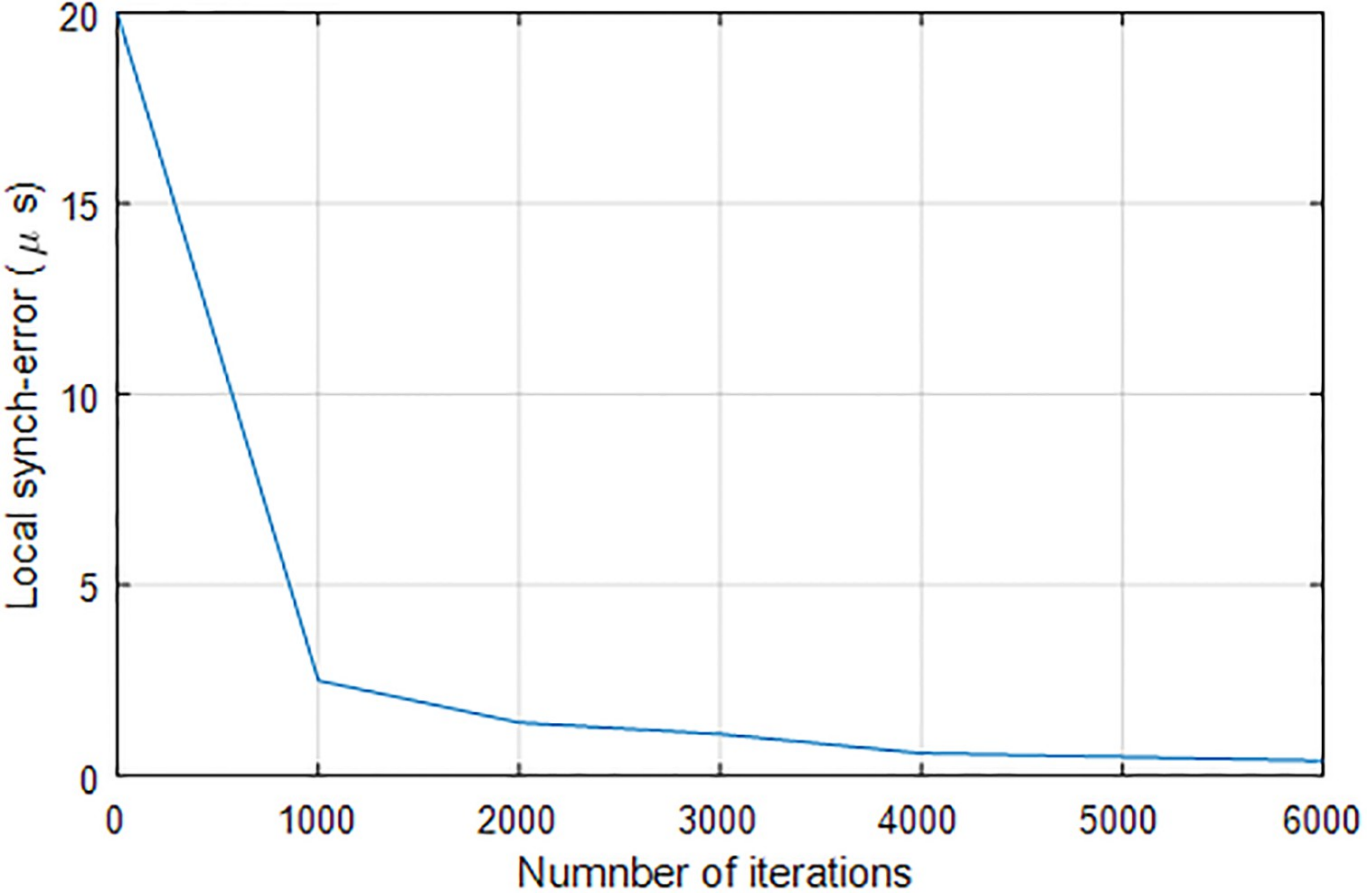
Convergence of algorithm.

Fig 16 illustrates the convergence of the proposed FGB algorithm under increasing number of iterations. As can be seen from Fig 16, the algorithm converges to local minimum that indicates its effectiveness.

### 6.9 Complexity analysis

**Fig 17:**
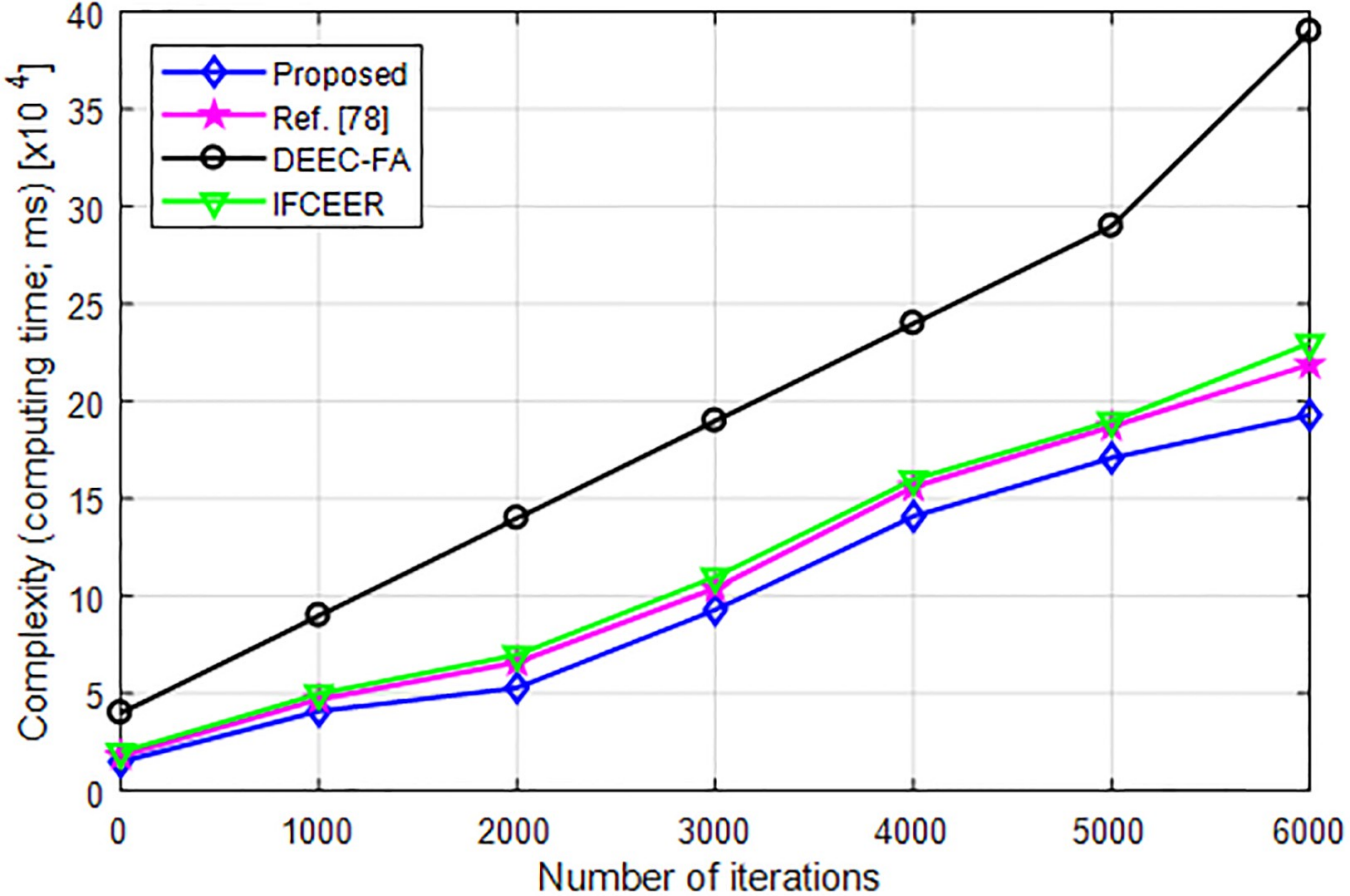
Computational complexity evaluation.

Fig 17 compares the computational complexity in terms of computing time of the proposed and existing algorithms with increasing number of iterations. As can be seen from Fig 17, the complexity of the proposed algorithm is lower than existing algorithms which makes is computationally efficient and easy to implement.

## 7 Conclusion and future work

The clustering routing technique for SDWSN is examined in this work. A hybrid optimization algorithm that combines the global optimization capabilities of FA with the local optimization capabilities of BBOGSA is developed with the goal of enhancing the performance of the optimization process for locating an approximate global solution. To hasten the rotation of cluster heads, a distributed high-efficiency entropy energy-saving clustering routing method is developed. The hybrid optimization method is compared with comparable algorithms in terms of convergence accuracy, running time, and stability through experimental simulation and analysis to demonstrate its optimization performance. To demonstrate how the distributed high-efficiency entropy energy-saving clustering routing algorithm may increase network longevity and decrease data transmission energy consumption, it is compared with comparable methods. The limitations of the proposed study are as follows that will be considered as next research. To increase the security of SDWSN, the following phase of development will look at creating a DDoS assault detection and defense system. It will employ an architecture that carries out multi-level DDoS mitigation in the data forwarding layer, first-level DDoS detection in the edge layer, second-level DDoS detection in the control layer, and clustering in the device layer. This approach will be more scalable, energy-efficient and improved.
